# Supplementary material for: Modeling Human Cytomegalovirus-Induced Microcephaly in Human iPSC-Derived Brain Organoids
Source: Cell Rep Med. 2020 Mar 25;1(1):100002. doi: 10.1016/j.xcrm.2020.100002 (PMC7659592; doi:10.1016/j.xcrm.2020.100002)
Supplement: Document S1. Figures S1–S7 [file mmc1.pdf]

Cell Reports Medicine, Volume *1*

Supplemental Information

Modeling Human Cytomegalovirus-Induced Microcephaly in Human iPSC-Derived Brain Organoids

Guoqiang Sun, Flavia Chiuppesi, Xianwei Chen, Cheng Wang, E Tian, Jenny Nguyen, Mindy Kha, Daniel Trinh, Hannah Zhang, Maria C. Marchetto, Hongjun Song, Guo-Li Ming, Fred H. Gage, Don J. Diamond, Felix Wussow, and Yanhong Shi

Supplemental Figures and Legends

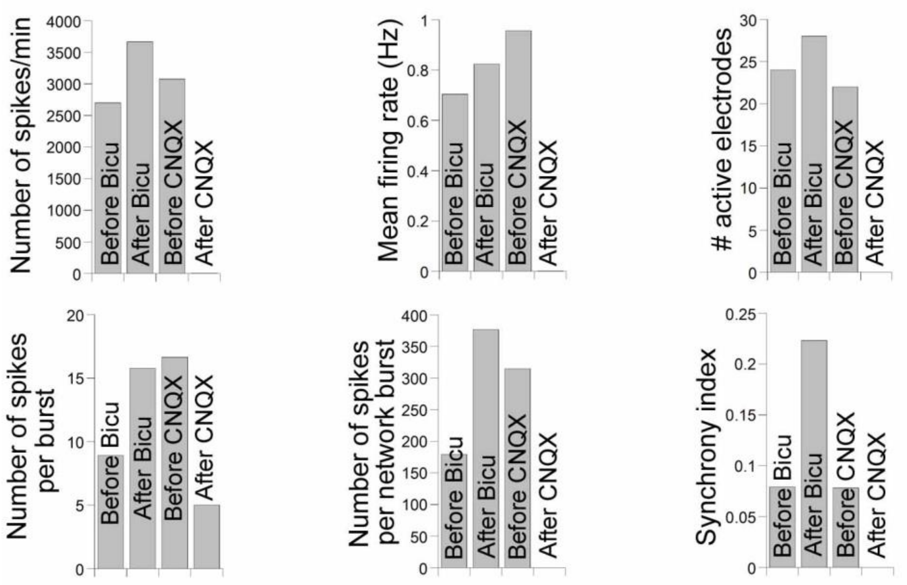

Figure S1. Functional neuronal networks shown by MEA recording. Related to Fig. 1. Quantification of MEA parameters in brain organoids in 10 min recording before or after treatment with GABAergic neuronal inhibitor bicuculine (Bicu), and before or after glutamatergic neuronal inhibitor CNQX treatments.

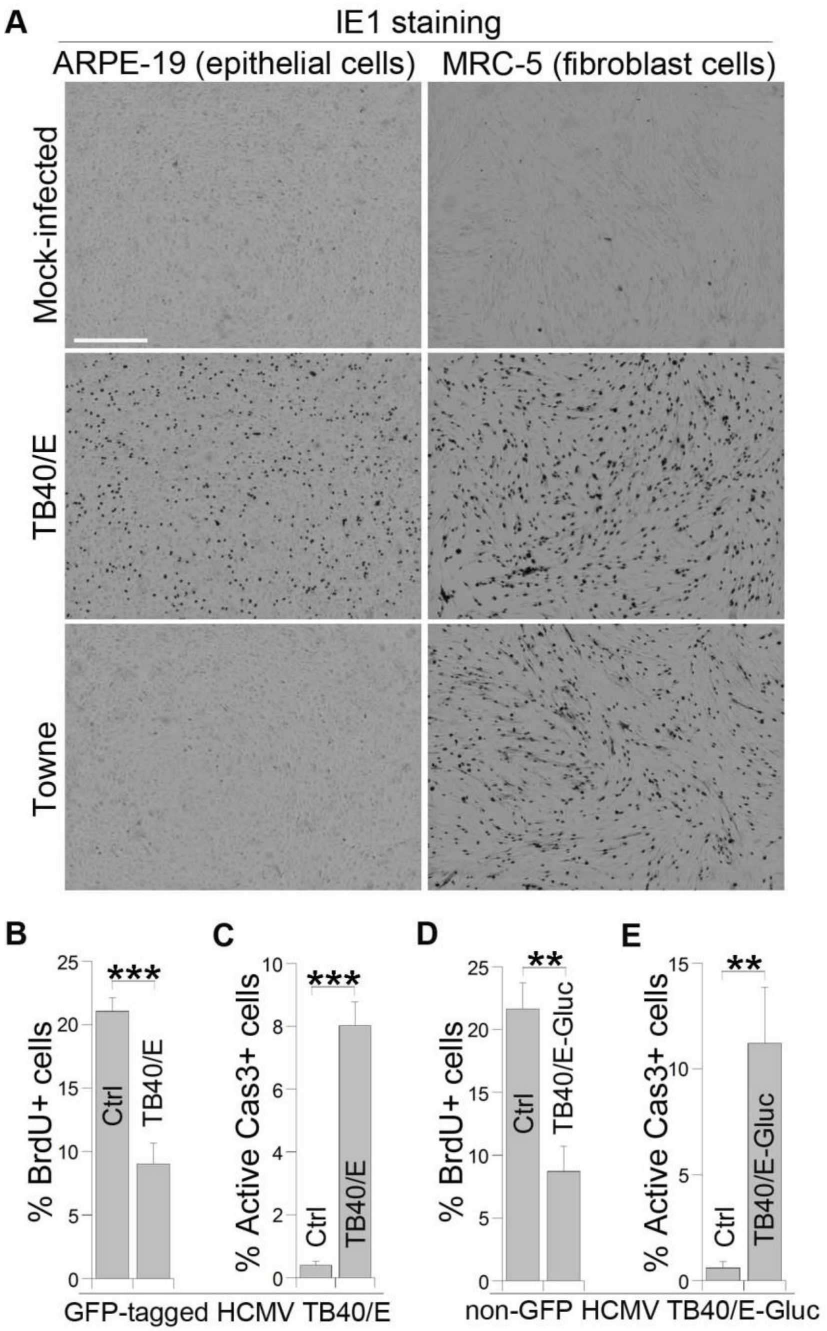

Figure S2. HCMV cell tropism and HCMV-induced abnormal cell proliferation and apoptosis in hiPSC-derived brain organoids. Related to Fig. 2. (**A**) ARPE-19 epithelial cells and MRC-5 fibroblasts were seeded in 96-well plates and either mock-infected or infected with TB40/E or Towne. HCMV-infected cells were immunostained for HCMV IE1 at 24 hours post infection. Shown are representative images of the IE1 immunostaining. Scale bar: 500 µm. (**B-E**) Quantification of the number of proliferating and apoptotic cells in brain organoids infected with the GFP-tagged HCMV TB40/E (B and C) or the non-GFP tagged HCMV TB40/E-Gluc (D and E) in comparison to mock-infected brain organoid controls. The number of proliferating cells was evaluated by the percent of BrdU+ cells out of total cells, while apoptosis was assessed by the percent of active caspase 3-positive (Active Cas3+) cells out of total cells. Bars represent mean ± SD. **$p<0.01$ and ***$p<0.001$ by Student's t-test . n=4 replicates.

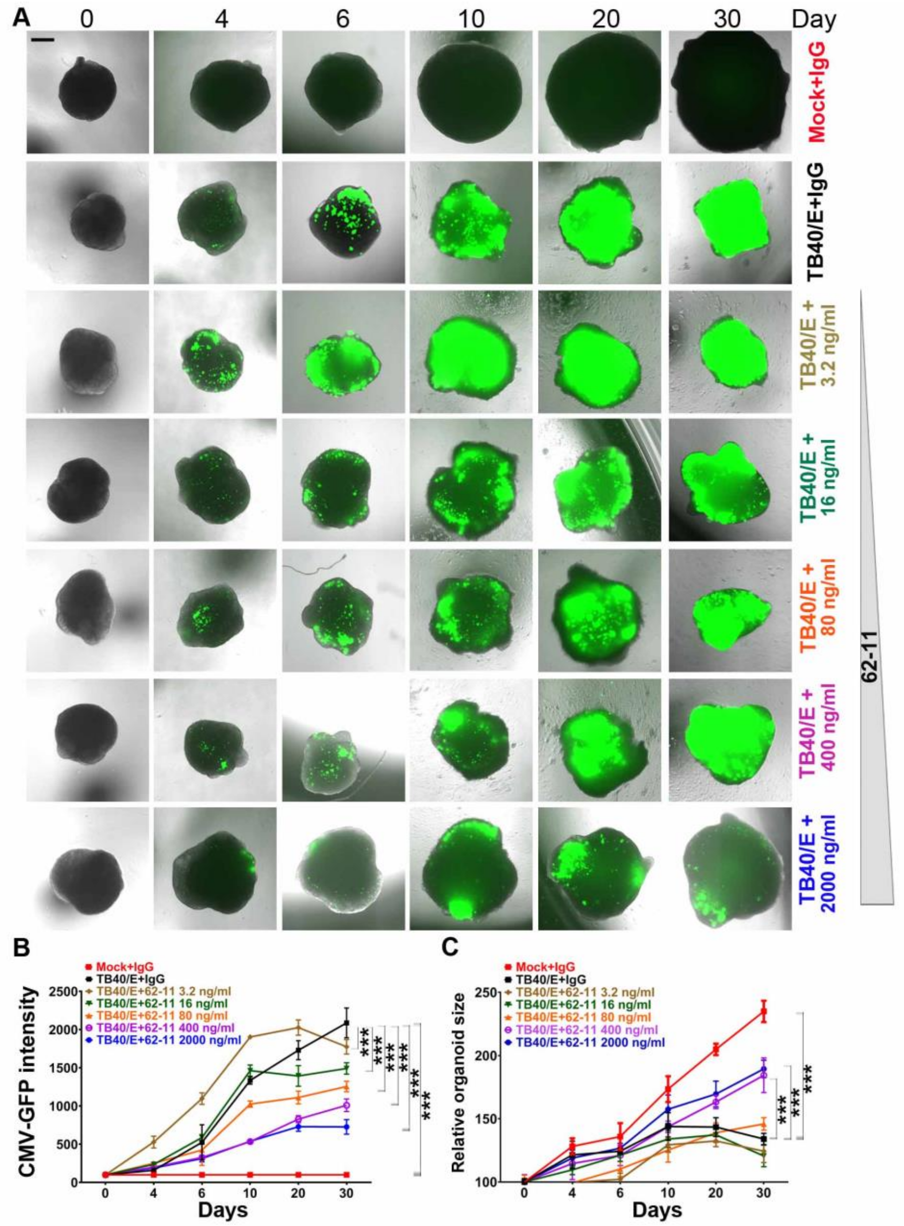

Figure S3. Prevention of HCMV TB40/E-induced abnormal brain organoid growth by Nab 62-11. Related to Fig. 3. hiPSC-derived brain organoids at day 45 of differentiation were infected with GFP-labeled TB40/E in the presence of different concentrations of NAb 62-11 that ranged from 3.2 ng/ml to 2000 ng/ml of antibody. Mock-infected brain organoids and TB40/E-infected organoids treated with IgG control (2000 ng/ml) were used as control organoids. **(A)** Representative images of control organoids and brain organoids infected with TB40/E in the presence of different concentrations of NAb 62-11. The images of the control organoids are the same as that in Fig. 3 because the experiments in Fig. 3 and Fig. S3 were performed in parallel. Scale bar, 200 µm. (**B, C)** Graphs illustrating the HCMV-GFP fluorescence intensity (B) and growth kinetics (C) of control organoids and TB40/E-infected brain organoids treated with NAb 62-11. Growth kinetics is measured using relative organoid size. The relative organoid size for each time point is given as the % of the organoid size (100%) at day 0 of infection. Values represent mean ± SD. *** p<0.001 by two way ANOVA followed by Tukey’s multiple comparison test. n=4 organoids per group.

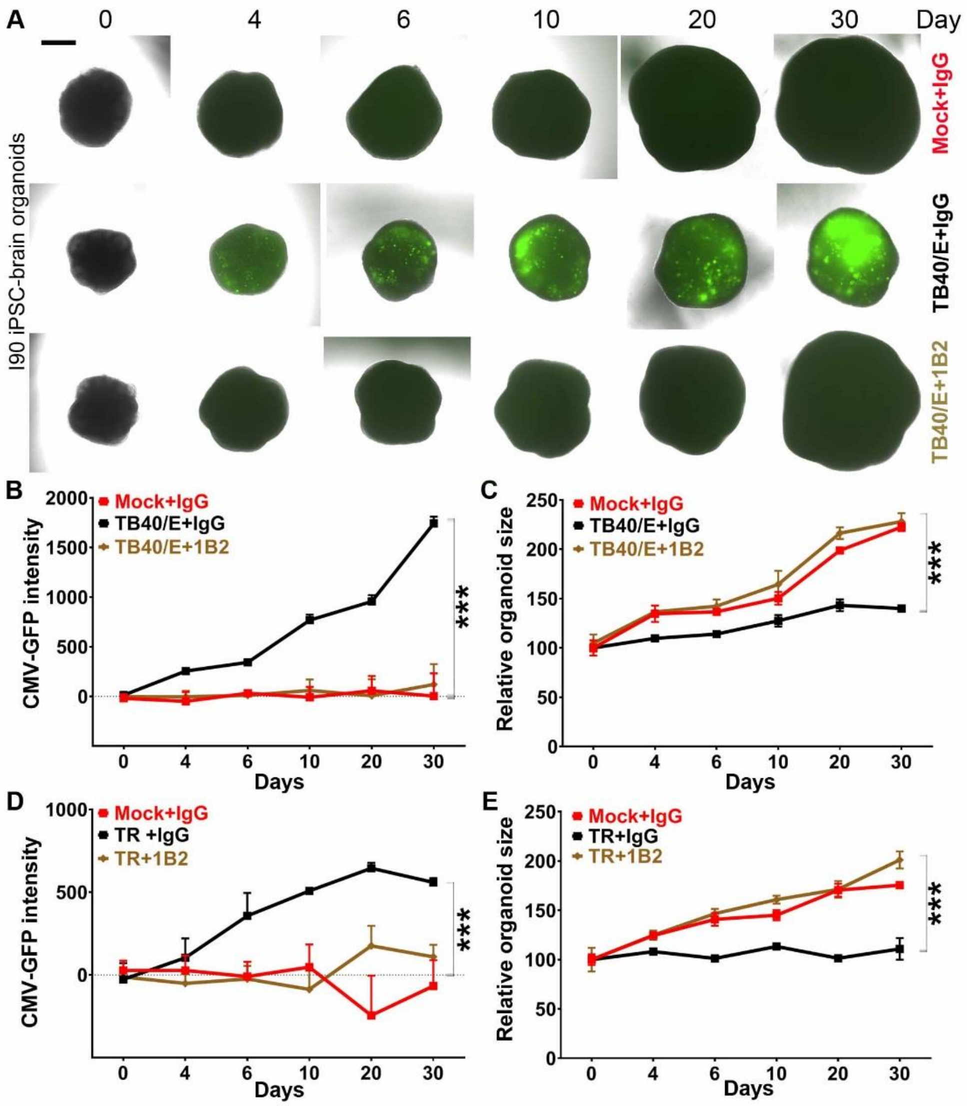

Figure S4. NAb prevents abnormal brain organoid growth induced by TB40/E and TR in hiPSC-derived organoids. Related to Fig. 3. Human IMR90 iPSC-derived brain organoids at day 45 of differentiation were infected with GFP-labeled TB40/E or TR in the absence or presence of NAb. Mock-infected brain organoids were used as a control. (**A**) Representative images of brain organoids that were mock-infected or infected with TB40/E in the presence of IgG (Mock + IgG, or TB40/E + IgG) or brain organoids infected with TB40/E in the presence of NAb 1B2 (TB40/E + 1B2) at the given time points during 30 days post infection. Scale bar, 200μm. (**B, C**) Graphs indicating the relative GFP fluorescence intensity (arbitrary units) (B) or relative organoid size (C) in mock-infected control brain organoids, or brain organoids infected with TB40/E in the absence or presence of NAb 1B2. (**D, E**) Graphs indicating the relative GFP fluorescence intensity (arbitrary units) (D) or relative organoid size (E) in mock-infected control brain organoids, or brain organoids infected with TR in the absence or presence of NAb 1B2. The relative organoid size for each time point is given as the % of the organoid size (100%) at day 0 of infection. Values represent mean ± SD. *** $p<0.001$ by two way ANOVA followed by Tukey's multiple comparison test. n=4 replicates.

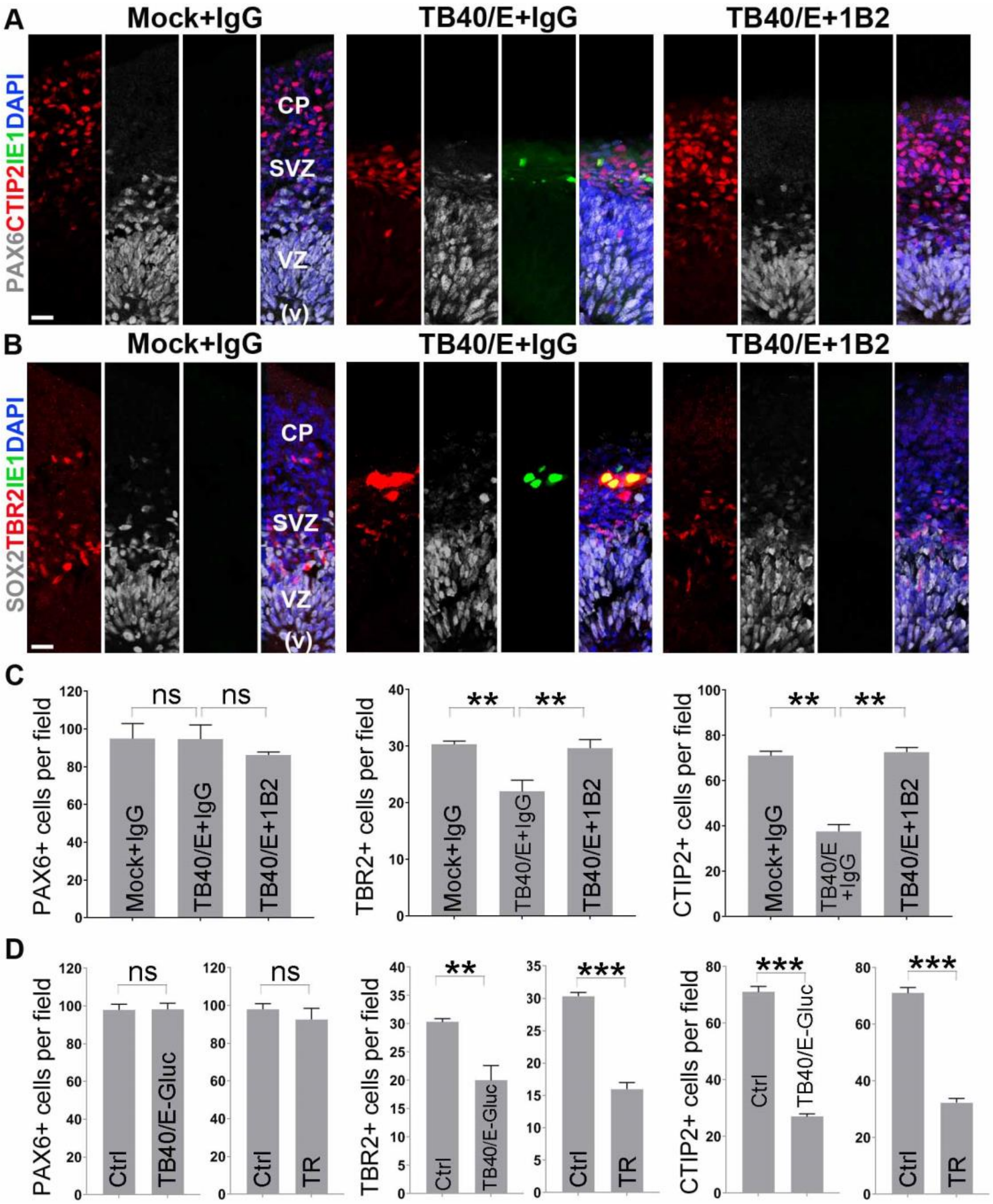

Figure S5. NAb prevents TB40/E-induced abnormal layer composition in brain organoids. Related to Fig. 4. (**A, B**) Representative images (cropped from images in Fig. 4C, D) were used for counting specific layer marker-positive cells. Scale bar, 20 µm. (**C**) Quantification of PAX6+, TBR2+, and CTIP2+ cells in brain organoids that were mock-infected in the presence of IgG control or infected with TB40/E in the presence of IgG control or NAb 1B2, in images shown in panels A and B. $p > 0.05$ (ns), $^{**}p < 0.01$ by one-way ANOVA followed by Tukey's multiple comparison test. n=4 replicates. (**D**) Quantification of PAX6+, TBR2+, and CTIP2+ cells in mock-infected control organoids and brain organoids infected with non-GFP-tagged TB40/E-Gluc or TR. $p > 0.05$ (ns), $^{**}p < 0.01$, $^{***}p < 0.001$ by Student's t-test. n=4 replicates. For panels C & D, values represent mean ± SD.

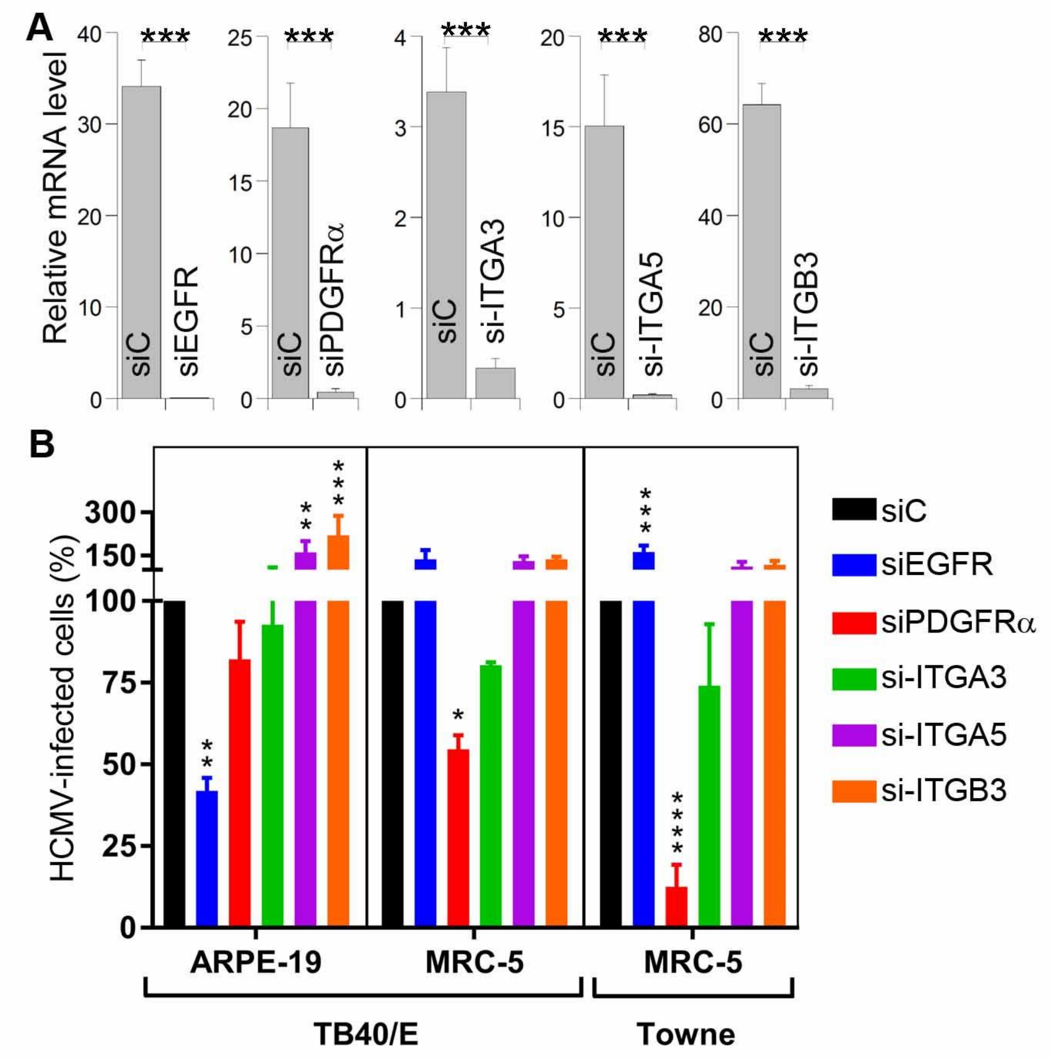

Figure S6. Knockdown of receptor gene expression by receptor-specific siRNAs. Related to Fig. 5. (A) Evaluation of siRNA efficacy. hiPSC-derived NPCs were transfected with siRNAs specific for PDGFRα, EGFR, ITGA5, ITGB3, ITGA3, or non-targeting control siRNA (siC). At four days post transfection, mRNA expression of specific genes was analyzed by RT-qPCR. Values represent mean ± SD. ***$p < 0.001$ by Student's t-test. n=4 replicates. (**B**) siRNA-mediated inhibition of HCMV infection of fibroblast and epithelial cells. ARPE-19 and MRC-5 cells were treated with the receptor-specific siRNAs or siC. Following 48-hour incubation, cells were infected with TB40/E or Towne and stained for HCMV IE1 at 24 hours post-infection. The percent of HCMV-infected cells was calculated relative to the number of IE1-positive cells in HCMV-infected cells treated with siC. Values represent mean ± SEM of three independent experiments performed in triplicate wells. Statistical significance was calculated to HCMV/siCTRL group using 2-way ANOVA followed by Dunnett's multiple comparisons test. *$p<0.05$, **$p<0.01$, ***$p<0.001$, and ****$p<0.0001$. n=3 replicates.

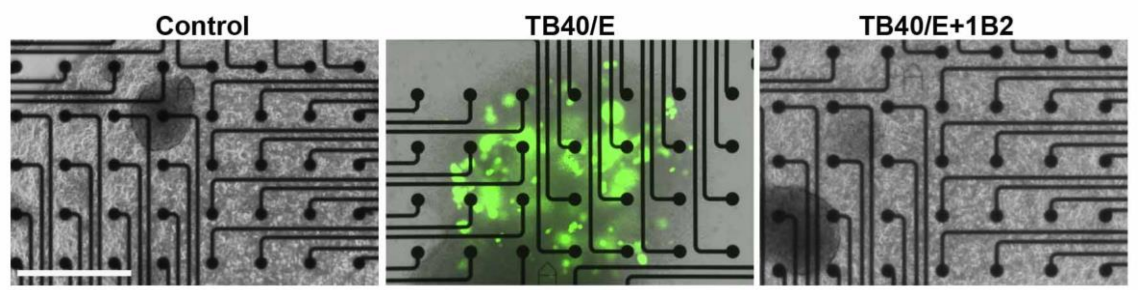

Figure S7. **Representative images of brain organoids seeded on MEA electrodes. Related to Fig. 7.** Sample images of mock-infected brain organoids and brain organoids infected with GFP-tagged TB40/E in the absence or presence of 1B2 NAb are shown. Scale: 500 µm.
