# Supplementary material for: Modeling Human Cytomegalovirus-Induced Microcephaly in Human iPSC-Derived Brain Organoids
Source: Cell Rep Med. 2020 Mar 25;1(1):100002. doi: 10.1016/j.xcrm.2020.100002 (PMC7659592; doi:10.1016/j.xcrm.2020.100002)
Supplement: Document S2. Article plus Supplemental Information [file mmc2.pdf]

Cell Reports
Medicine

Article

Modeling Human Cytomegalovirus-Induced Microcephaly in Human iPSC-Derived Brain Organoids

Graphical Abstract

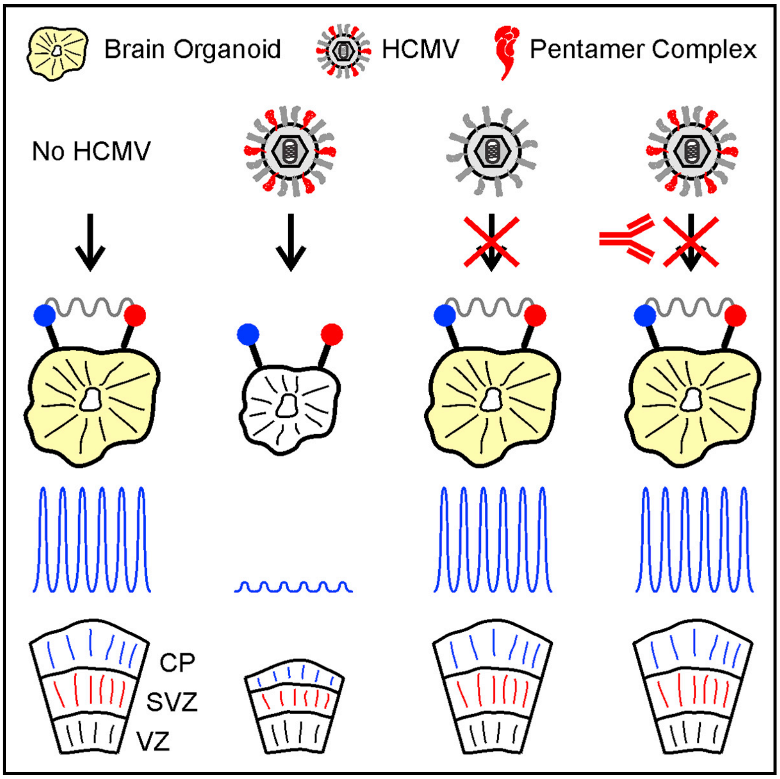

Highlights

- Human iPSC-derived brain organoids to model HCMV-induced brain malformation
- "Clinical-like" HCMV strain impairs human brain organoid growth and structure
- HCMV-infected brain organoids exhibit abnormal calcium signaling and neural network
- HCMV-induced brain organoid abnormality can be prevented by neutralizing antibodies

Authors

Guoqiang Sun, Flavia Chiuppesi, Xianwei Chen, ..., Don J. Diamond, Felix Wussow, Yanhong Shi

Correspondence

fwussow@coh.org (F.W.), yshi@coh.org (Y.S.)

In Brief

Sun et al. used human-iPSC-derived brain organoids to model the effects of HCMV infection on human brain development. They found that a "clinical-like" HCMV strain impairs brain organoid growth, structure, and neural network activity. Moreover, the abnormal brain organoid development caused by HCMV can be prevented by neutralizing antibodies.

Sun et al., 2020, Cell Reports Medicine *1*, 100002
April 21, 2020 © 2020 The Authors.
https://doi.org/10.1016/j.xcrm.2020.100002

Cell Reports Medicine

CellPress
OPEN ACCESS

Article

Modeling Human Cytomegalovirus-Induced Microcephaly in Human iPSC-Derived Brain Organoids

Guoqiang Sun,[1,5] Flavia Chiuppesi,[2,5] Xianwei Chen,[1,5] Cheng Wang,[1] E Tian,[1] Jenny Nguyen,[2] Mindy Kha,[2] Daniel Trinh,[1] Hannah Zhang,[1] Maria C. Marchetto,[3] Hongjun Song,[4] Guo-Li Ming,[4] Fred H. Gage,[3] Don J. Diamond,[2] Felix Wussow,[2,*] and Yanhong Shi[1,6,*]

[1]Division of Stem Cell Biology Research, Department of Developmental and Stem Cell Biology, Beckman Research Institute of City of Hope, 1500 E. Duarte Road, Duarte, CA 91010, USA
[2]Department of Hematology, Beckman Research Institute of City of Hope, 1500 E. Duarte Road, Duarte, CA 91010, USA
[3]Laboratory of Genetics, Salk Institute for Biological Studies, 10010 North Torrey Pines Road, San Diego, CA 92037, USA
[4]Department of Neuroscience and Mahoney Institute for Neurosciences and Department of Cell and Developmental Biology, Institute for Regenerative Medicine, University of Pennsylvania School of Medicine, Philadelphia, PA 19104, USA
[5]These authors contributed equally
[6]Lead Contact
*Correspondence: fwussow@coh.org (F.W.), yshi@coh.org (Y.S.)
https://doi.org/10.1016/j.xcrm.2020.100002

SUMMARY

Although congenital infection by human cytomegalovirus (HCMV) is well recognized as a leading cause of neurodevelopmental defects, HCMV neuropathogenesis remains poorly understood. A major challenge for investigating HCMV-induced abnormal brain development is the strict CMV species specificity, which prevents the use of animal models to directly study brain defects caused by HCMV. We show that infection of human-induced pluripotent-stem-cell-derived brain organoids by a "clinical-like" HCMV strain results in reduced brain organoid growth, impaired formation of cortical layers, and abnormal calcium signaling and neural network activity. Moreover, we show that the impeded brain organoid development caused by HCMV can be prevented by neutralizing antibodies (NAbs) that recognize the HCMV pentamer complex. These results demonstrate in a three-dimensional cellular biosystem that HCMV can impair the development and function of the human brain and provide insights into the potential capacity of NAbs to mitigate brain defects resulted from HCMV infection.

INTRODUCTION

Human cytomegalovirus (HCMV) is a ubiquitous and highly adapted human pathogen that establishes lifelong latency in infected individuals. Although it is usually benign, HCMV infection during pregnancy can result in viral transmission to the developing fetus, thereby causing irreparable birth defects in newborns.[1,2] Congenital HCMV infection can occur following primary maternal infection or result from non-primary maternal infection due to maternal re-infection or viral reactivation.[3,4] It is estimated that 0.5%–2% of all newborns worldwide are affected by congenital HCMV infection.[5,6] Around 10%–15% of congenitally infected newborns are symptomatic at birth, and these infants may suffer from neurodevelopmental deficits, including hearing loss, intellectual disability, microcephaly, or cerebral palsy.[2,7,8] In addition, congenitally infected newborns that are asymptomatic at birth may develop neurological illness later in life.[2,7] In the United States, children with long-term medical conditions are estimated to be more frequently associated with congenital HCMV infection than with other well-known childhood diseases, such as Down syndrome, fetal alcohol syndrome, and spina bifida.[9]

Despite the recognition of congenital HCMV infection as a leading cause of neurological defects in newborns, HCMV-induced neuropathogenesis remains poorly understood.[2] Although studies with mouse and rhesus CMV in their respective animal models have been used to gain insights into HCMV-induced brain malformation,[2,10] these surrogate animal models may not faithfully address the neuropathology of HCMV. The strict CMV species specificity limits studies of HCMV infection to *in vitro* cell culture system and excludes the use of animals to directly investigate HCMV-specific brain disorders or to identify antiviral strategies that could mitigate neurological defects specifically associated with HCMV infection.[2]

Human induced pluripotent stem cell (hiPSC)-derived brain organoids have emerged as a powerful *in vitro* model to study human brain development and neurodevelopmental diseases in a three-dimensional (3D) cellular biosystem.[11-14] These so-called "mini brains" can organize into discrete and interdependent brain regions that are reminiscent of the human cortex

This is an open access article under the CC BY license (http://creativecommons.org/licenses/by/4.0/).

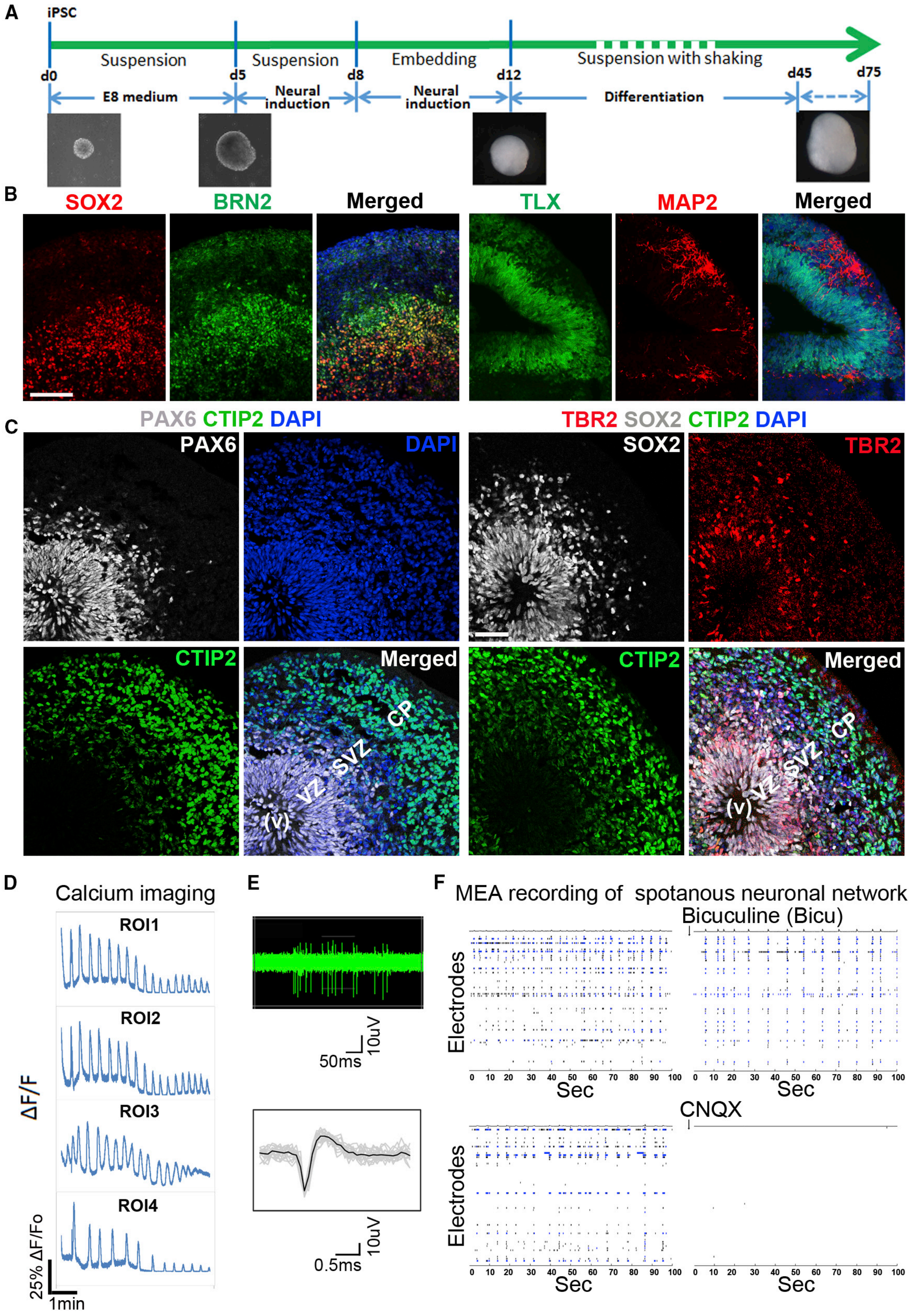

(legend on next page)

and form multilayered structures containing inner neural progenitor zones and outer cortical layers.[15] Brain organoids have been used to model neurological defects caused by Zika virus,[14] which has been linked to microcephaly.[16,17]

In this study, we generated brain organoids from hiPSCs to model brain defects caused by HCMV infection *in vitro* in a 3D human cellular biosystem. We infected hiPSC-derived brain organoids with the "clinical-like" HCMV strain TB40/E to determine how HCMV infection impacts early human brain development, determined potential cellular receptors that mediate brain cell infection by HCMV, and tested the capacity of neutralizing antibodies (NAbs) to prevent HCMV-induced brain malformation.

RESULTS

Generation and Characterization of hiPSC-Derived Brain Organoids

In order to study the effects of HCMV infection on early human brain development, we generated hiPSC-derived brain organoids using a protocol similar to that described by Lancaster et al.[15,18] and characterized the brain organoids at day 45–75 of differentiation (Figure 1A). This stage of hiPSC-derived brain organoids has been shown to mimic human fetal brain development at the transition between the first and second trimester of gestation.[19] The hiPSC-derived brain organoids developed into layered structures containing a core region positive for the neural progenitor markers SOX2 and TLX[20] and an outer layer positive for BRN2, a late-born superficial layer neuronal marker, and MAP2, a marker for mature neurons (Figure 1B). Further characterization revealed the presence of the PAX6-positive apical progenitors, the cell body of which primarily locates in the ventricular zone (VZ), and the TBR2-positive basal progenitors, the cell body of which mostly resides in the subventricular zone (SVZ) (Figure 1C).[21] We also detected the SOX2-positive progenitors that span across the VZ and SVZ and the CTIP2-positive neurons that reside mainly in a region corresponding to the cortical plate (CP) (Figure 1C). Calcium imaging of selected brain organoid regions revealed multiple synchronized calcium surges upon stimulation with glutamate (Figure 1D). These results suggest the presence of mature and active neurons in hiPSC-derived brain organoids. Further characterization by multielectrode arrays (MEAs) showed that the brain organoids formed a neuronal network with spontaneous, synchronized burst and spikes, which could be quenched by the glutamatergic neuron blocker CNQX, but not by the GABAergic neuron blocker bicuculline (Figures 1E, 1F, and S1).[22] This result indicates that the neurons within the human brain organoids could form an electrophysiologically active neuronal network, which is characteristic of the development of interdependent cortical layers in the developing human brain.[23,24] These results together indicate that hiPSC-derived brain organoids could recapitulate aspects of early human brain development.

HCMV TB40/E Impairs the Growth and Cortical Structure of hiPSC-Derived Brain Organoids

Using recombinant viruses of HCMV strains TB40/E and Towne that express a GFP reporter,[25,26] we evaluated the consequences of HCMV infection on the development of hiPSC-derived brain organoids. In contrast to the laboratory HCMV strain Towne, the clinical-like HCMV strain TB40/E expresses the envelope pentamer complex (PC) and was able to efficiently infect both fibroblasts and epithelial cells, whereas Towne was only able to efficiently infect fibroblast, but not epithelial, cells (Figure S2A). These observations are consistent with previous findings.[27-30]

To determine the effect of HCMV infection on brain organoid growth, hiPSC-derived brain organoids at day 45 of differentiation were exposed to GFP-labeled TB40/E or Towne virus. The GFP fluorescence intensity and the brain organoid size were evaluated over a period of 20 days after viral exposure. Mock-infected brain organoids were used as a negative control. We found that the clinical-like HCMV strain TB40/E could efficiently infect and propagate in the brain organoids and severely compromise the brain organoid growth, as revealed by an increase in GFP fluorescence intensity in the infected organoids and substantially reduced size of the infected brain organoids, when compared to the mock-infected brain organoid controls (Figures 2A–2C). In contrast, the laboratory HCMV strain Towne did not efficiently infect human brain organoids and therefore did not significantly impair the growth of brain organoids (Figures 2A–2C). These results indicate that the clinical-like HCMV strain TB40/E is able to infect human brain organoids and trigger reduced size of brain organoids, mimicking HCMV-induced microcephaly. Because the clinical-like HCMV strain TB40/E is PC proficient, whereas the laboratory HCMV strain Towne is PC deficient, the difference in infection efficiency by TB40/E and Towne in human brain organoids suggests that efficient HCMV infection of human brain organoids is dependent on the PC.

Figure 1. Generation of hiPSC-Derived Brain Organoids

(A) A schematic illustration of brain organoid derivation from hiPSCs.

(B and C) Characterization of brain organoids by marker staining. Immunostaining hiPSC-derived brain organoids at day 45 (B) and day 75 (C) of differentiation for the neural progenitor markers SOX2 and TLX (B) and PAX6, SOX2, and TBR2 (C) and the neuronal markers BRN2 and MAP2 (B) and CTIP2 (C) are shown. The merged images include DAPI staining (blue). CP, cortical plate; SVZ, subventricular zone V, ventricle; VZ, ventricle zone. Scale bars, 100 μm.

(D) Calcium imaging of brain organoids at day 45 of differentiation. Single-cell tracing of calcium surge upon glutamate stimulation in selected regions of interest (ROIs) is shown. The change in fluorescence intensity (arbitrary units) of the calcium dye was plotted over time.

(E) Voltage waveforms of brain organoids. Spontaneous activity recorded from a single electrode of MEA for 6 s in a brain organoid at 75 days of differentiation is shown (top panel). The waveform of all detected spikes from top panel is shown in gray, and the mean waveform is shown in black (bottom panel).

(F) MEA recording of brain organoids. Graphs show a representative MEA recording generated from the raw data of a spike raster plot, using the number of spikes recorded over 100 s. The top panels show MEA recording of hiPSC-derived brain organoids before (left panel) or after (right panel) treatment with the GABAergic neuronal inhibitor bicuculline. The bottom panels show MEA recording of hiPSC-derived brain organoid before (left panel) or after (right panel) treatment with the glutamatergic neuronal inhibitor CNQX.

See also Figure S1.

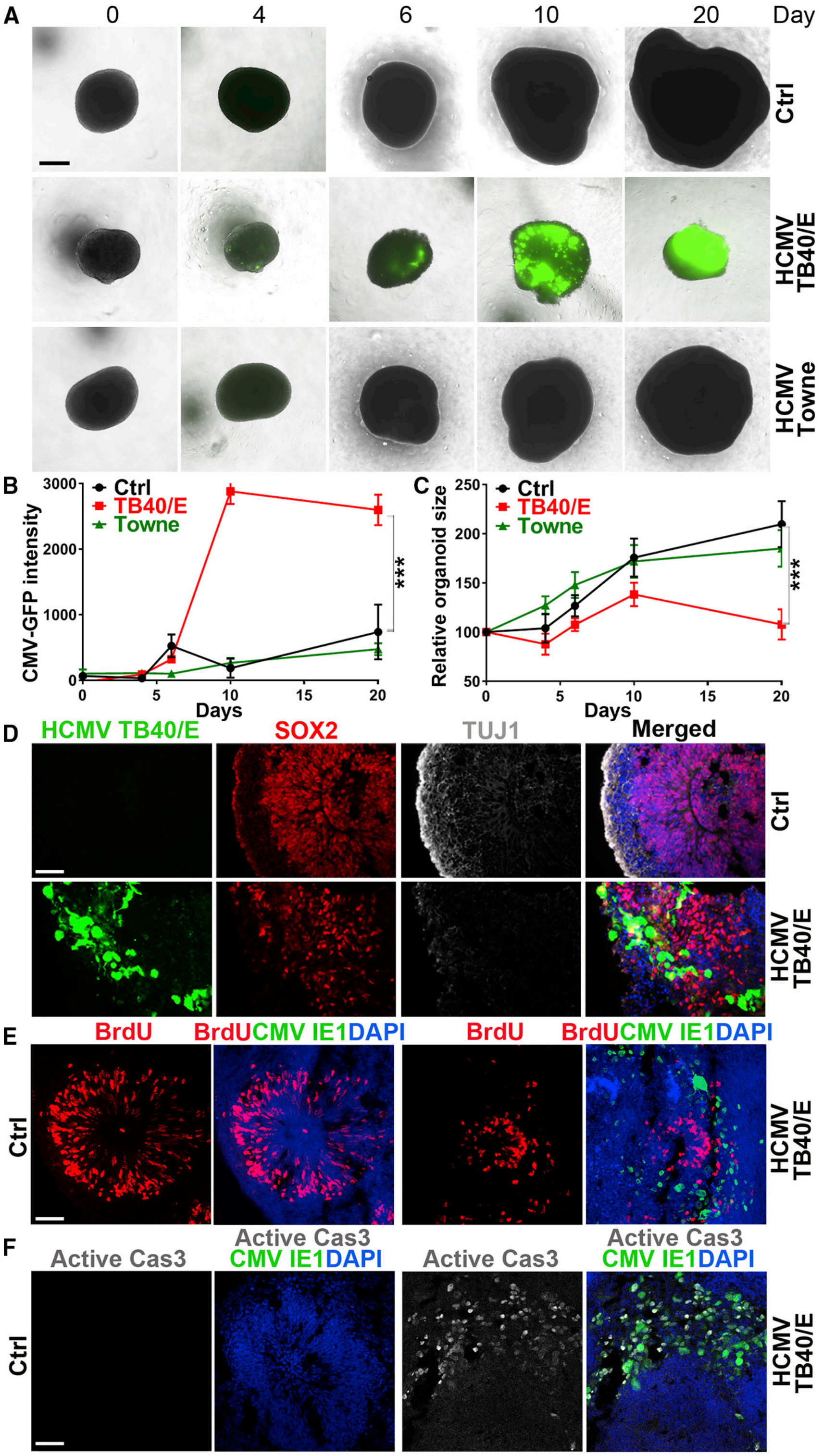

Figure 2. HCMV TB40/E Impairs hiPSC-Derived Brain Organoid Development

(A–C) Inhibition of brain organoid growth by HCMV TB40/E. hiPSC-derived brain organoids at day 45 of differentiation were infected with GFP-labeled TB40/E or Towne. Mock-infected brain organoids were used as a control. Immunofluorescence images of the mock-infected (Ctrl) and TB40/E- or Towne-infected brain organoids at the designated days post-infection are shown in (A). Scale bar, 200 μm. Graphs indicating the relative GFP fluorescence intensity (arbitrary units) or relative organoid size in HCMV-infected brain organoids versus mock-infected control brain organoids are shown in (B) and (C). Growth kinetics was measured using relative organoid size. The relative organoid size for each time point is given as the % of day 0 organoid size in diameter (100%).

(D) Impaired layer structure in TB40/E-infected brain organoids. Brain organoids were mock-infected (Ctrl) or infected with TB40/E at day 30 of differentiation and then stained for the neural progenitor marker SOX2 or the neuronal marker TUJ1 at day 15 post-infection (day 45 of differentiation). Scale bar, 20 μm.

(E and F) Decreased cell proliferation and increased apoptosis in TB40/E-infected brain organoids. Brain organoids were infected with TB40/E at day 45 of differentiation. Brain organoids were mock-infected (Ctrl) or infected with TB40/E at day 30 of differentiation. The number of proliferating and apoptotic cells was determined by BrdU labeling (E) or immunostaining for active caspase 3 (Cas3) (F). HCMV-infected cells were labeled by staining for HCMV IE1. Scale bars, 50 μm.

For (B) and (C), values represent mean ± SD. ***$p < 0.001$ by two-way ANOVA followed by Tukey's multiple comparison test. n = 4 organoids per group. See also Figure S2.

Further characterization of the mock-infected and TB40/E-infected brain organoids 15 days after infection revealed that TB40/E compromised the formation of the outer layer of the SOX2-positive progenitor core and the TUJ1-positive neuronal layer in human brain organoids (Figure 2D), indicating that TB40/E impairs the formation of outer neural progenitor and cortical layers in the infected brain organoids. In addition, staining with the proliferative marker bromodeoxyuridine (BrdU) and the apoptotic marker active caspase 3 (Cas3) revealed a decrease in the number of proliferating cells and an increase in the number of apoptotic cells in TB40/E-infected brain organoids when compared to the control organoids (Figures 2E, 2F, S2B, and S2C). In a parallel study, we infected the hiPSC-derived brain organoids with a non-GFP-tagged virus variant of TB40/E, termed TB40/E-Gluc.[31] As with the GFP-tagged TB40/E, the non-GFP-tagged TB40/E-Gluc caused a decrease in the number of proliferating cells and an increase in apoptotic cells in infected organoids (Figures S2B–S2E). These results together indicate that the clinical-like HCMV strain TB40/E can efficiently infect human brain

CellPress
OPEN ACCESS

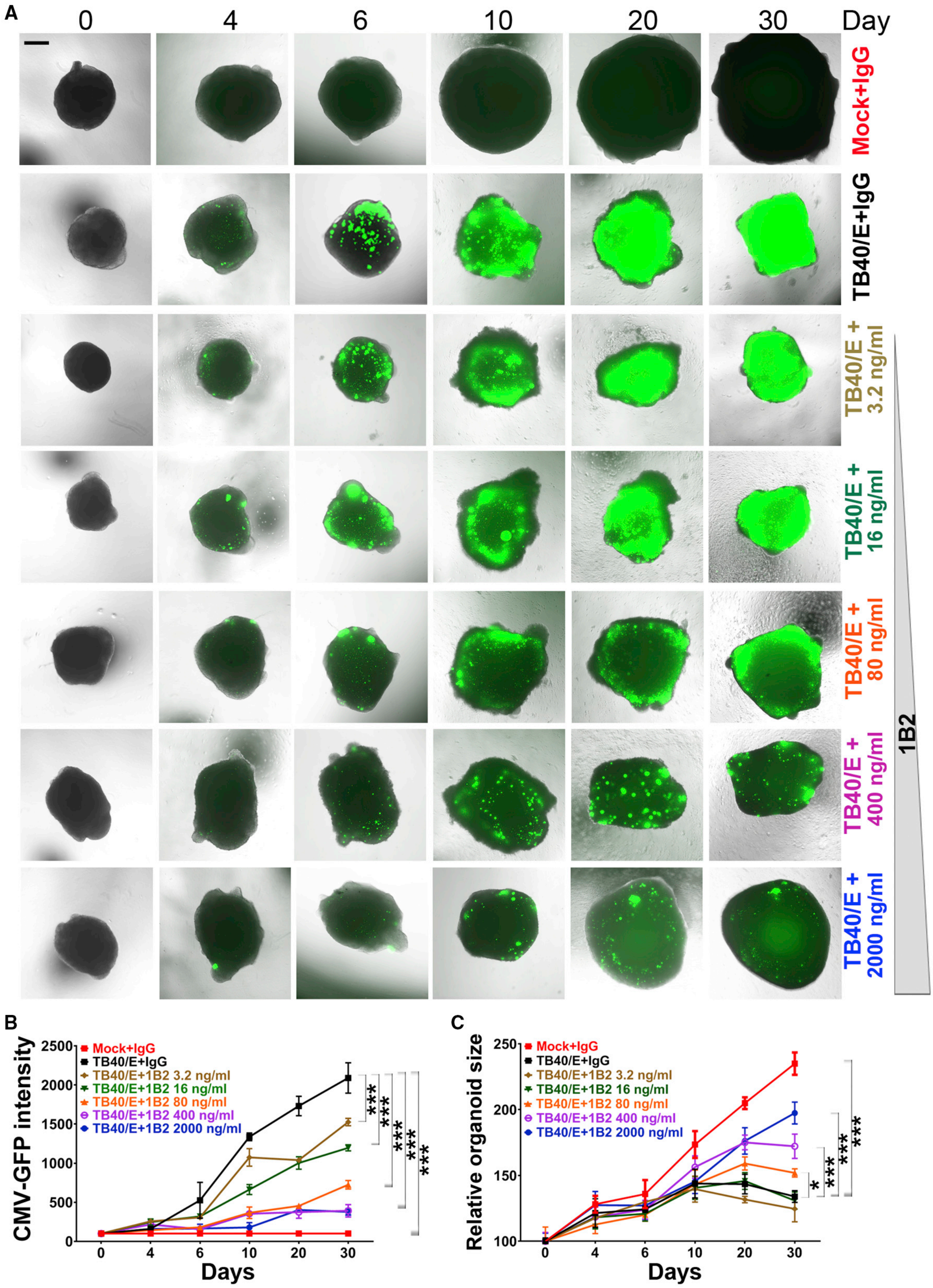

(legend on next page)

organoids and impairs the growth and structure of hiPSC-derived brain organoids.

NAbs Effectively Prevent HCMV TB40/E-Induced Abnormal Brain Organoid Growth and Structure

Although NAbs are considered important for preventing congenital HCMV infection,[32-35] their capacity to interfere with HCMV infection of the developing human brain remains unknown. To gain insight into the potential of NAbs to interfere with HCMV infection of human brain cells, we evaluated the prevention of TB40/E infection of hiPSC-derived brain organoids by two previously isolated NAbs, 1B2 and 62-11, that were raised in mice against the envelope PC by vaccination.[36,37] NAb 1B2 is an antibody that targets the UL128/130/131A subunits of the PC. 1B2 is unable to prevent HCMV infection of fibroblasts but confers exceptionally high potency to inhibit HCMV infection of epithelial cells.[36] In contrast, NAb 62-11 is a gH-specific antibody that can potently interfere with both fibroblast and epithelial cell infection.[36,37]

Using the GFP-labeled TB40/E virus, we infected human brain organoids at day 45 of differentiation in the presence of different concentrations of the 1B2 or 62-11 antibody (3.2–2,000 ng/mL) and measured GFP fluorescence intensity and brain organoid size for up to 30 days post-infection. Mock-infected and TB40/E-infected brain organoids treated with immunoglobulin G (IgG) control were used as control organoids. Compared to brain organoids infected with TB40/E in the presence of IgG control, organoids infected with TB40/E in the presence of either the 1B2 or 62-11 NAb exhibited substantially reduced GFP fluorescence intensity and markedly increased growth kinetics in a dose-dependent manner. More dramatic effects were observed with higher concentrations of the 1B2 or 62-11 NAb (Figures 3 and S3). Brain organoids infected with TB40/E in the presence of 80–2,000 ng/mL of the 1B2 or 62-11 antibody were substantially larger than organoids infected with TB40/E in the presence of IgG control (Figures 3, S3, and S4A). To determine whether infection with other clinical-like HCMV strains besides TB40/E could also induce a microcephaly-like phenotype, we infected hiPSC-derived brain organoids with another clinical-like HCMV strain, termed TR.[38] As observed with TB40/E, we found substantially reduced brain organoid size following infection with TR (Figures S4B–S4E). Moreover, the infection of brain organoids by TR and its effect on brain organoid growth could be prevented by NAb 1B2 (Figures S4D and S4E). These results indicate that PC-specific NAbs can inhibit brain organoid infection by the clinical-like HCMV strains TB40/E and TR, thereby allowing normal brain organoid growth.

In addition to preventing brain organoid growth restriction by TB40/E, the NAb treatment also inhibited abnormal cortical structure formation induced by TB40/E. Cortical layers in brain organoids were defined by layer-specific marker staining (Figure 4A). Immunostaining for HCMV immediate-early 1 (IE1) protein revealed that the HCMV-positive cells were predominantly found in the TBR2$^+$ SVZ (Figure 4B). Accordingly, when compared to the mock-infected organoids treated with IgG control, the TB40/E-infected brain organoids treated with the IgG control displayed substantially reduced thickness of the TBR2$^+$ SVZ and the CTIP2$^+$ CP layers, although the PAX6$^+$ VZ layer was not considerably affected (Figures 4C, 4D, and 4G). In contrast, the TBR2$^+$ SVZ and the CTIP2$^+$ CP layers in the brain organoids infected with TB40/E in the presence of the 1B2 antibody were much thicker than those in organoids treated with TB40/E in the presence of the IgG control, similar to those of the mock-infected brain organoids treated with the IgG control (Figures 4C, 4D, and 4G). The number of the TBR2$^+$ cells and the CTIP2$^+$ cells in these organoids was changed accordingly (Figures S5A–S5C). A similar effect on the TBR2$^+$ and the CTIP2$^+$ layer thickness was observed when brain organoids were infected with non-GFP-tagged TB40/E-Gluc or TR (Figure S5D). Further characterization of the infected brain organoids with markers for cortical-layer-specific neurons revealed reduced thicknesses of both deep and superficial cortical layers in organoids infected with TB40/E in the presence of IgG control when compared to mock-infected organoids treated with IgG control. In contrast, the cortical layers in the organoids infected with TB40/E in the presence of 1B2 NAb were similar to those of the mock-infected organoids treated with the IgG control (Figures 4E, 4F, and 4H). These results together indicate that NAbs targeting the epitopes of HCMV PC can potently prevent HCMV infection of human brain organoids, thus allowing normal brain organoid growth and cortical layer formation.

PDGFRα and EGFR Are Involved in HCMV TB40/E Infection of Human Brain Organoids

To support the relevance of the brain organoid model to study HCMV infection of the developing human brain, we investigated potential cellular receptors that are involved in brain organoid infection by TB40/E. Several receptors, such as platelet-derived growth factor receptor alpha (PDGFRα), epidermal growth factor receptor (EGFR), and integrins (α3, α5, and β3), have been previously described to be either directly or indirectly involved in HCMV host cell entry.[39-41]

To determine the importance of these cellular receptors for brain organoid infection by TB40/E, we knocked down the

Figure 3. Prevention of HCMV TB40/E-Induced Abnormal Brain Organoid Growth by NAbs

hiPSC-derived brain organoids at day 45 of differentiation were infected with GFP-labeled TB40/E in the presence of different concentrations of NAb 1B2 that ranged from 3.2 ng/mL to 2,000 ng/mL. Mock-infected and TB40/E-infected brain organoids in the presence of IgG control (2,000 ng/mL) were used as controls.

(A) Representative images of control organoids and brain organoids infected with TB40/E in the presence of different concentrations of 1B2 antibody. Scale bar, 200 μm.

(B and C) Graphs illustrating the CMV-GFP fluorescence intensity (B) and growth kinetics (C) of control organoids and organoids infected with TB40/E in the presence of different concentrations of NAb 1B2. Growth kinetics was measured using relative organoid size. The relative organoid size for each time point is given as the % of the organoid size (100%) at day 0 of infection. Values represent mean ± SD. ***$p < 0.001$ by two-way ANOVA followed by Tukey's multiple comparison test. n = 4 organoids per group.

See also Figures S3 and S4.

CellPress
OPEN ACCESS

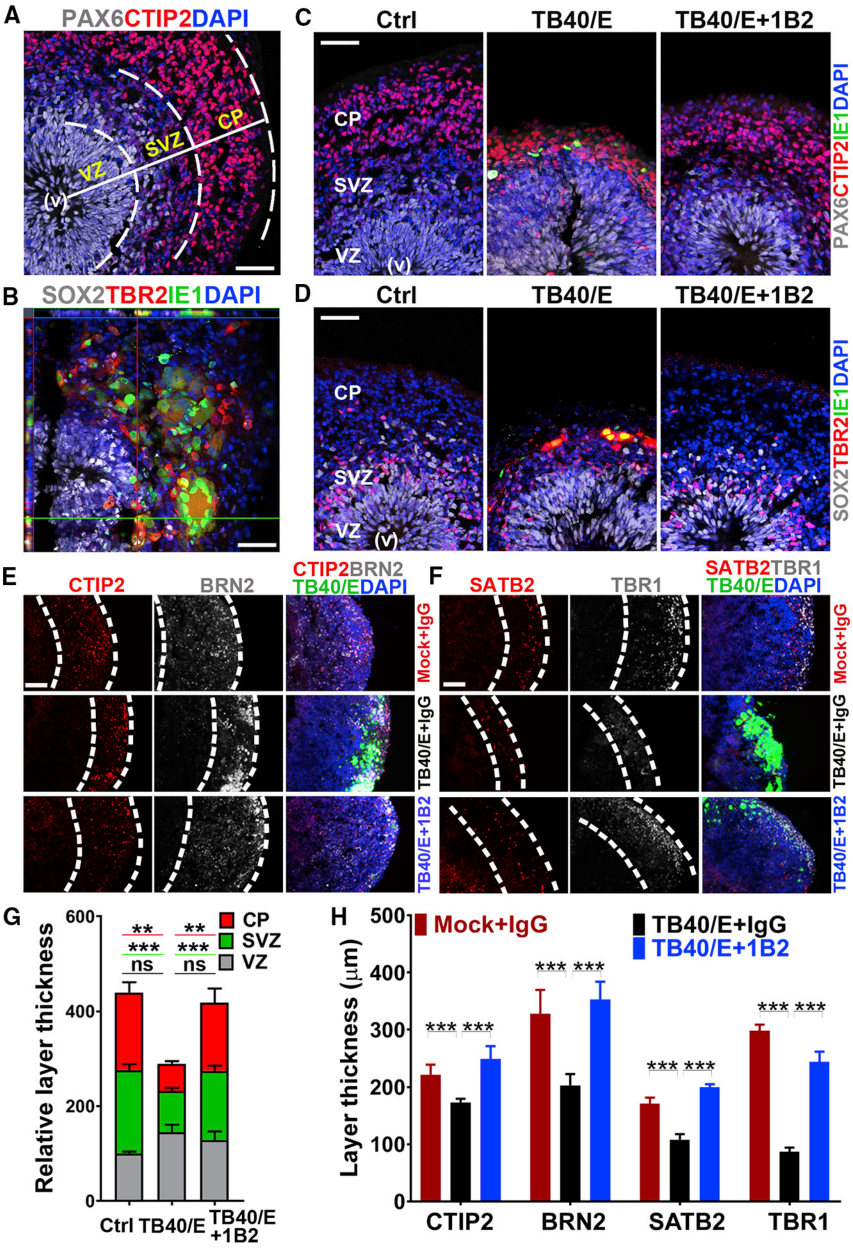

Figure 4. NAb-Mediated Prevention of TB40/E-Induced Abnormal Brain Organoid Structure

(A) A representative image showing layer specification of the VZ, SVZ, and CP in brain organoids at day 75 of differentiation.

(B) An orthogonal view of HCMV-infected brain organoids stained for the HCMV IE1 and the progenitor markers SOX2 and TBR2.

(C and D) Representative images of brain organoids stained for the progenitor markers PAX6 (C), SOX2 and TBR2 (D), the neuronal marker CTIP2, and HCMV IE1. Brain organoids were mock infected or infected with TB40/E in the presence of IgG control antibody as organoid controls or infected with TB40/E in the presence NAb 1B2 (2,000 ng/mL).

(E and F) Representative images of brain organoids stained for different neuronal markers. Control brain organoid and brain organoids infected with TB40/E in the presence of NAb 1B2 were stained for the cortical neuronal markers CTIP2 and BRN2 (E) and SATB2 and TBR1 (F) at day 30 post-infection.

(G) Graph comparing the relative layer thicknesses (%) of the VZ, SVZ, and CP in control organoids and brain organoids infected with TB40/E in the presence of 1B2 antibody. The relative thickness is normalized to Ctrl VZ.

(H) Graph comparing the layer thicknesses of the CTIP2-, BRN2-, SATB2-, or TBR1-positive layers in control organoids and brain organoids treated with TB40/E in the presence of 1B2 antibody.

Scale bars, 50 μm for (A)–(D), 100 μm for (E) and (F). Values represent mean ± SD. $^{**}p < 0.01$; $^{***}p < 0.001$ by ANOVA followed by Tukey's multiple comparison test. n = 4 organoids per group. See also Figure S5.

expression of individual receptors using receptor-specific small interfering RNAs (siRNAs). The knockdown efficiency of each receptor was verified in hiPSC-derived neural progenitor cells (NPCs) by qRT-PCR (Figure S6A). We first confirmed the differential involvement of EGFR and PDGFRα in HCMV entry into epithelial cells and fibroblasts, respectively (Figure S6B).[42] We then treated hiPSC-derived brain organoids at day 45 of differentiation with different receptor-specific siRNA or a control siRNA for 4 days and subsequently exposed the organoids to the GFP-labeled HCMV TB40/E. Following HCMV exposure, the GFP fluorescence intensity and the brain organoid size was evaluated for up to 20 days. Brain organoids treated with the integrin α3 (ITGA3), α5 (ITGA5), or β3 (ITGB3)-specific siRNA exhibited similar GFP fluorescence intensity and growth kinetics to those of control siRNA-treated brain organoids (Figures 5A–5C). In contrast, brain organoids treated with the EGFR- or PDGFRα-specific siRNA showed no or very low GFP fluorescence during the entire 20-day post-infection period and exhibited substantially higher growth kinetics compared to control siRNA-treated organoids (Figures 5A–5C). These results indicate that both PDGFRα and EGFR are involved in HCMV infection of the brain organoids. Western blot analysis revealed that both EGFR and PDGFRα were expressed at higher levels in NPCs than neurons (Figure 5D). To support the importance of these receptors in HCMV infection, we evaluated whether overexpression of these receptors in HCMV-resistant cells could render these cells susceptible to HCMV infection. We prepared EGFR and PDGFRα expression vectors and confirmed their expression by western blot (Figure 5E). Using these vectors, we found that overexpression of EGFR and PDGFRα in hiPSCs, which are resistant to

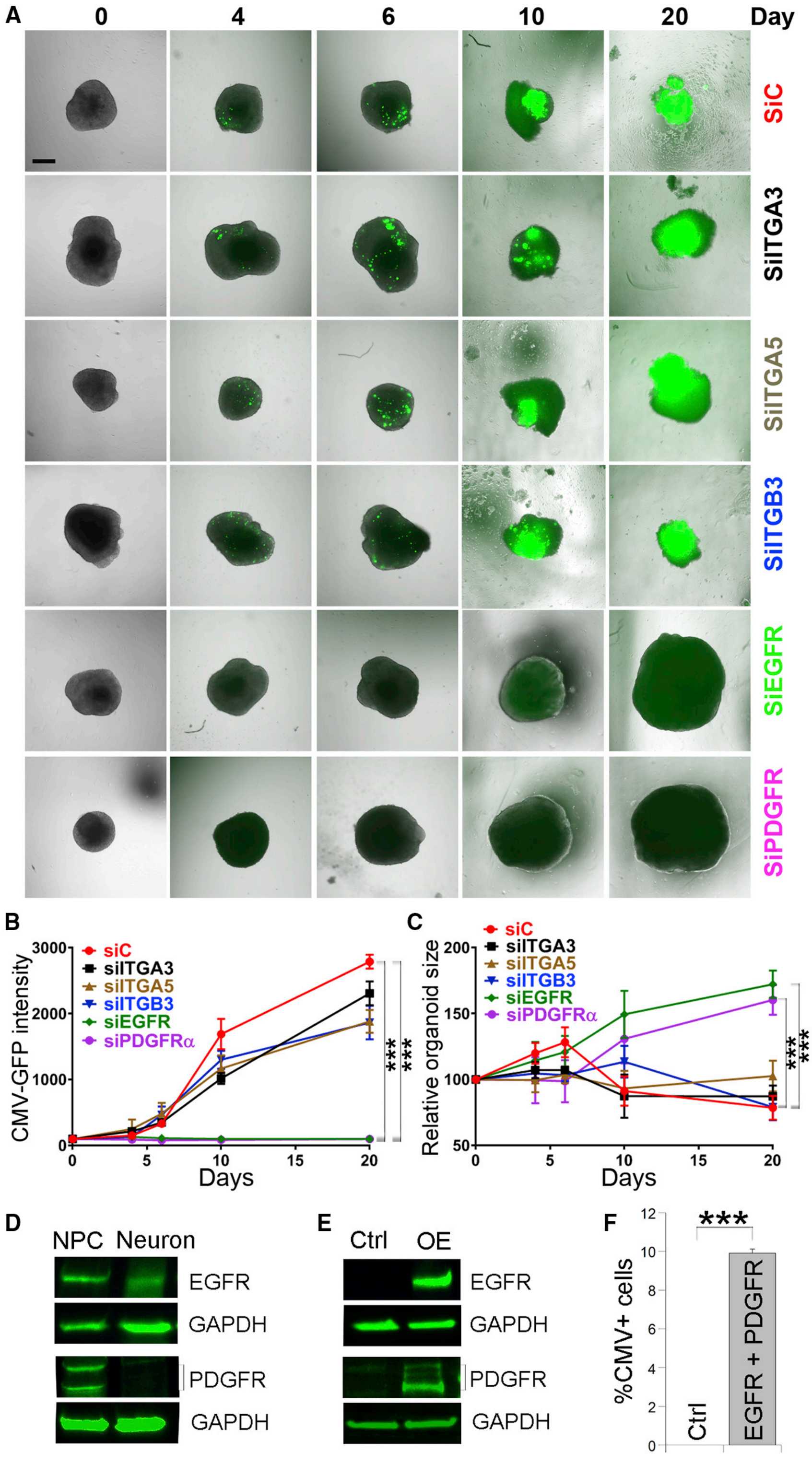

Figure 5. EGFR and PDGFRα Are Involved in TB40/E Infection of Brain Organoids

hiPSC-derived brain organoids at day 45 of differentiation were treated with siRNAs specific for EGFR; PDGFRα; integrin α3, α5, or β3; or control siRNA for 4 days and subsequently infected with GFP-labeled TB40/E.

(A) Immunofluorescence images of TB40/E-infected brain organoids pre-treated with different siRNAs. Scale bar, 500 μm.

(B and C) Graphs illustrating the HCMV GFP fluorescence intensity (B) and brain organoid growth kinetics (C). The relative organoid sizes for each time point are given as the % of the organoid size (100%) at day 0 of infection. Values represent mean ± SD. ***$p < 0.001$ by two-way ANOVA followed by Tukey's multiple comparison test. n = 4 organoids per group.

(D) Western blot analysis of the expression of EGFR and PDGFRα in hiPSC-derived NPCs and neurons. GAPDH was included as a loading control.

(E) Western blot analysis showing overexpression (OE) of EGFR and PDGFRα in HEK293T cells. GAPDH was included as a loading control.

(F) Overexpression of EGFR and PDGFRα renders hiPSCs susceptible to HCMV infection. hiPSCs were electroporated with a control vector expressing RFP or the combination of vectors expressing RFP together with EGFR and PDGFRα and subsequently infected with HCMV TB40/E. The HCMV-infected cells were stained by the HCMV marker IE1. The percent of HCMV-positive cells was quantified by the percent of CMV IE1-positive (CMV^{+}) cells out of total cells. Values represent mean ± SD. ***$p < 0.001$ by Student's t test. n = 4 replicates.

See also Figure S6.

HCMV infection,[43] rendered these cells permissive to TB40/E infection (Figure 5F), supporting the idea that both EGFR and PDGFRα are important mediators of HCMV infection. These results together indicate that HCMV infection of human brain organoids involves both EGFR and PDGFRα, whereas it does not appear to depend on integrins, such as α3, α5, or β3 integrin.

HCMV TB40/E Disrupts Calcium Signaling and Neural Network Activity in Brain Organoids

In order to uncover mechanisms of HCMV-induced neurodevelopmental defects, we performed RNA sequencing analysis in control and HCMV TB40/E-infected brain organoids. Interestingly, we found that at least three out of the top ten downregulated genes in HCMV TB40/E-infected organoids were related to calcium signaling (Figure 6A). These genes include ENO2, a neuron-specific enolase that could bind to calcium;[44] BNIP3, a gene involved in endoplasmic reticulum (ER)/mitochondria Ca^{2+} homeostasis;[45] and PDK1, a gene involved in regulation of Ca^{2+} entry into cells (Figure 6A).[46] Gene ontology (GO) analysis revealed that genes significantly downregulated in TB40/E-infected brain organoids include those involved in neurodevelopment, including brain development, astrocyte

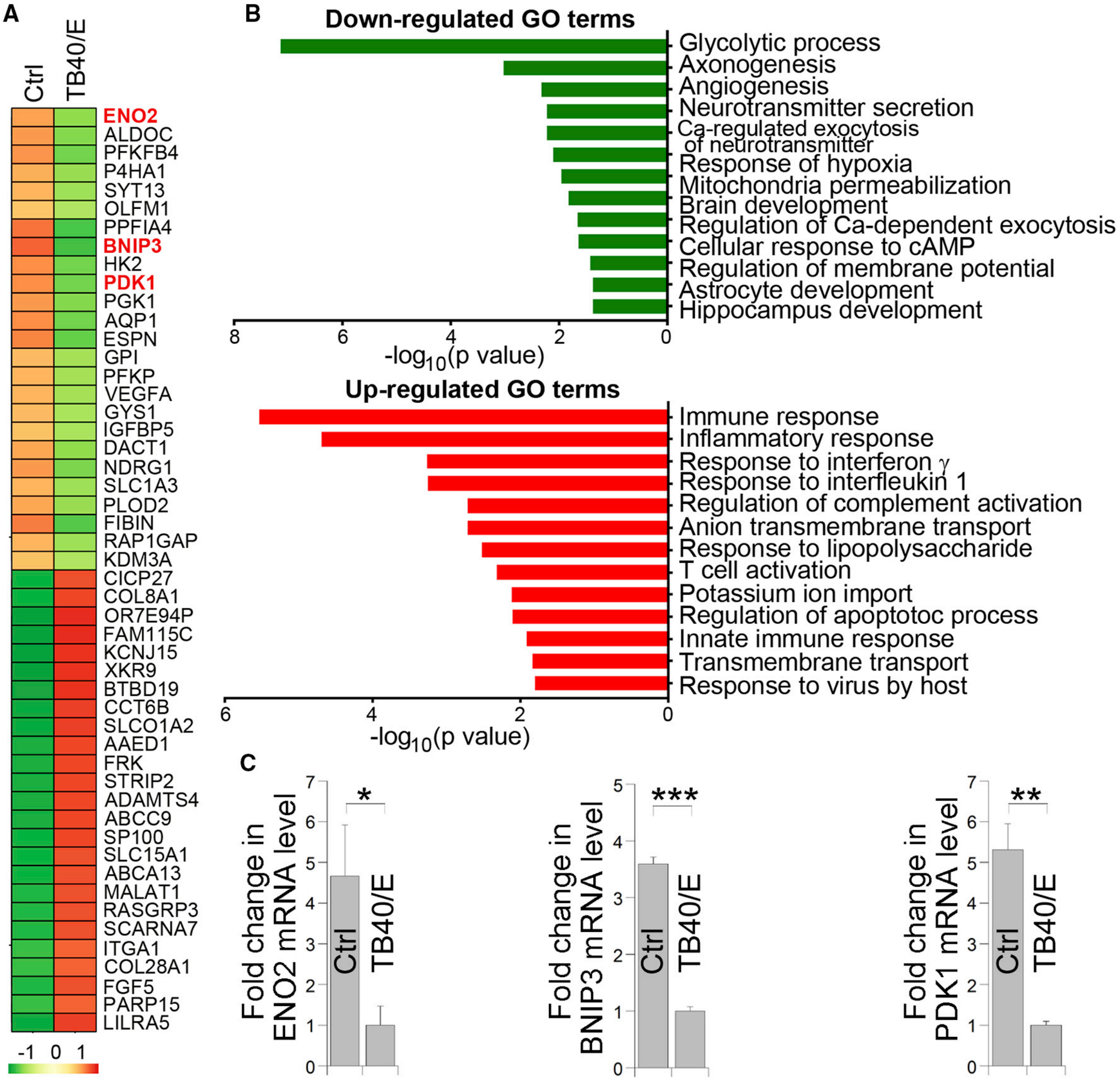

Figure 6. Calcium Signaling Is Affected in HCMV-Infected Brain Organoids

RNA sequencing analysis of control and TB40/E-infected brain organoids. Brain organoids at day 45 differentiation were infected with TB40/E and analyzed by RNA sequencing analysis 15 days post-infection.

(A) Heatmap summary of changes in mRNA expression levels in TB40/E-infected brain organoids versus UV-irradiated TB40/E-infected control brain organoids (Ctrl).

(B) GO analysis of down- and upregulated genes in TB40/E-infected brain organoids, compared control organoids, ranked by $-\log_{10}$ (p value).

(C) qRT-PCR analysis of selected downregulated genes, ENO2, BNIP3, and PDK1, in TB40/E-infected brain organoids. Values represent mean ± SD. *p < 0.05, **p < 0.01, and ***p < 0.001 by Student's t test. n = 4 replicates.

development, and hippocampal development, and genes implicated in calcium-regulated exocytosis of neurotransmitter and regulation of calcium-dependent exocytosis (Figure 6B). On the other hand, genes significantly upregulated in TB40/E-infected brain organoids include those involved in immune response and inflammatory response (Figure 6B). qRT-PCR analysis confirmed decreased expression of ENO2, BNIP3, and PDK1, three calcium-signaling-related genes, in TB40/E-infected brain organoids (Figure 6C).

The downregulation of genes involved in calcium signaling in TB40/E-treated brain organoids prompted us to test whether calcium response is impaired in HCMV-infected organoids. Using calcium imaging, we measured Ca^{2+} oscillations in control brain organoids and TB40/E-treated brain organoids, as well as brain organoids infected with TB40/E in the presence of 1B2 NAb. Because the calcium signal was monitored using a green fluorescent calcium dye, we used TB40/E virus without a GFP reporter to infect human brain organoids, and the HCMV-infected cells were detected by immunostaining for the HCMV IE1 protein. Although control brain organoids exhibited frequent bursts of calcium surges, no calcium activity was detected in HCMV-positive regions (identified by $IE1^+$ staining) in HCMV-

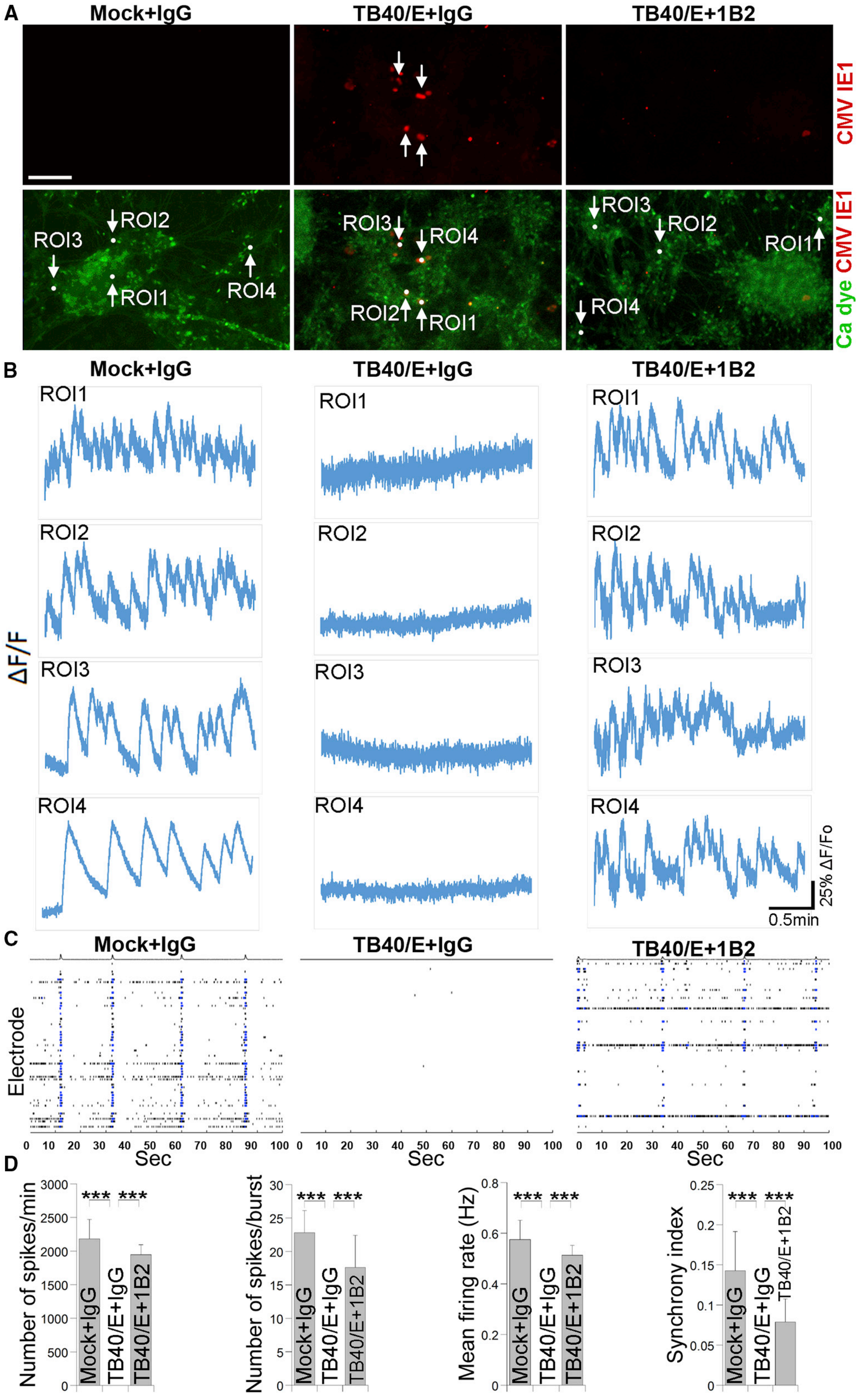

(legend on next page)

infected brain organoids (Figures 7A and 7B). In contrast, the brain organoids infected with TB40/E in the presence of 1B2 NAb displayed bursts of calcium surges that were in frequency and magnitude similar to those of the control organoids (Figures 7A and 7B). These results indicate that HCMV TB40/E abrogates calcium signaling in infected brain organoids, which can be prevented by anti-HCMV PC NAbs.

In addition to measuring neural activity using calcium imaging, we also evaluated neural network activity in TB40/E-infected brain organoids by MEA (Figure S7). We were able to detect multiple synchronized bursts and spikes by MEA recording in control brain organoids (Figures 7C and 7D). In contrast, TB40/E-infected brain organoids displayed significantly reduced neural network activity, including reduced number of spikes, mean firing rate, and synchrony index (Figures 7C and 7D). Consistent with the finding from calcium imaging, treatment with the 1B2 NAb largely prevented neural network impairment induced by TB40/E, resulting in an MEA profile similar to that of the control brain organoids (Figures 7C and 7D). These results together indicate that HCMV can severely impair neural network activity in infected brain organoids, which can be largely prevented by the treatment with a HCMV PC-specific NAb.

DISCUSSION

One of the main limitations in modeling HCMV-induced neurodevelopmental disorders is the lack of an *in vitro* model that could faithfully recapitulate HCMV infection of the developing human brain. Here, we have used hiPSC-derived brain organoids to model effects of HCMV infection on early human brain development in a 3D human brain organoid system. We have shown that the clinical-like HCMV strain TB40/E can efficiently infect hiPSC-derived brain organoids and propagate in these organoids, thereby impairing brain organoid growth and cortical structure, a phenotype reminiscent of HCMV-induced microcephaly. Moreover, we demonstrate that HCMV infection of human brain organoids can result in drastically abnormal calcium signaling and neural network activity. Of interest, we found that the neurodevelopmental defects induced by TB40/E in infected brain organoids can be largely prevented by NAbs that target the HCMV PC, a primary target of protective antibody responses and pivotal component of current subunit vaccine designs to prevent intrauterine HCMV transmission.[47] After initial submission of this paper, two other research articles have been published that describe effects of HCMV infection on early human brain development using human brain organoid models.[48,49]

To model the clinical effects of HCMV infection on early human brain development, we used hiPSC-derived brain organoids at day 45 of differentiation.[15,18] This stage of hiPSC-derived brain organoids is reminiscent of early human fetal brain development at the transition between the first and second trimester,[19] when congenital HCMV infection is known to be associated with severe neurological consequences.[7,50] Our observation that HCMV can severely impair the growth, structure, and neural network activity of hiPSC-derived brain organoids provides a possible explanation for HCMV-induced neurological defects during early gestation.[2] Our data appear consistent with the clinical manifestation of HCMV-induced microcephaly, including abnormal cortical development.[8,51] Clinical data for neural network activity or brain electrophysiology in patients congenitally infected with HCMV are limited.[2] Our findings in TB40/E-infected brain organoids may therefore provide novel insights into the dramatic consequences that congenital HCMV infection could have on neural network formation during early human brain development.

In humans, HCMV-immune-labeled cells can be found ubiquitously distributed in the ventricular zone, subventricular zone, and cortical plate in the brains of congenitally infected fetuses.[8] Although HCMV can target different cell types in the brains of congenitally infected fetuses, it shows higher tropism to neural progenitor cells.[8] We have shown that HCMV primarily infects the $TBR2^+$ cells in the SVZ of the brain organoids and that HCMV infection of the brain organoids disrupts the organization of the SVZ and the formation of proper cortical layers, suggesting that HCMV targets predominantly neural progenitors in the SVZ, thereby impairing cortical development. HCMV infection at early stage of human brain development could dramatically affect the expansion of the neocortex and consequently lead to reduced brain size and impaired brain function. Although the brain organoid model may provide a direct link between HCMV infection of the human brain and the development of microcephaly, severe neurological conditions due to congenital HCMV infection may also result from HCMV-induced placental insufficiency, which could lead to nutrition or oxygen deprivation in the fetal compartment.[2]

To support the relevance of the brain organoid model to study HCMV infection of the developing human brain, we evaluated potential cellular receptors that are involved in brain organoid infection by TB40/E. Consistent with recent findings for HCMV

Figure 7. HCMV Infection Disrupts Calcium Signaling and Neural Network Activity in Brain Organoids

(A and B) Calcium imaging of HCMV-infected brain organoids. Brain organoids at day 45 of differentiation were infected with HCMV TB40/E (without GFP reporter) in the presence of IgG control antibody or NAb 1B2 and analyzed by calcium imaging 4 days post-infection. Brain organoids infected with UV-irradiated TB40/E (mock infected) in the presence of IgG control antibody were included as a control.

(A) Representative images of HCMV IE1 immunostaining (red) are shown at the top panel and of Ca^{2+} dye fluorescence (green) are shown at the bottom panel. Scale bar, 200 μm.

(B) Four regions of interest (ROIs) were selected, and the Ca^{2+} dye fluorescence intensity graph of the ROIs is shown.

(C) MEA analysis of HCMV-infected brain organoids. Brain organoids at day 45 of differentiation were infected with TB40/E in the presence of IgG control antibody or NAb 1B2, and MEA recording was performed 2 weeks post-infection. Brain organoids infected with UV-irradiated TB40/E (mock infected) in the presence of IgG control were used as a control. Graphs illustrate MEA recording generated from the raw data of a spike raster plot, using the number of spikes recorded over 100 s.

(D) Quantification of the MEA parameters measured in (C). Values represent mean ± SD. ***$p < 0.001$ by Student's t test. n = 3 independent wells in MEA plates. See also Figure S7.

entry,[39,40,42,52] we have shown that TB40/E infection of human brain organoid involves PDGFRα and EGFR as cellular receptors either directly or indirectly, whereas the infection does not appear to involve cellular integrins. Although PDGFRα has been shown to function as a cellular receptor during HCMV entry into fibroblasts via interaction with a trimeric complex composed of gH/gL/gO,[42,53,54] EGFR is thought to be involved in PC-mediated entry into epithelial cells.[42,55] We found that both PDGFRα and EGFR are important for TB40/E infection of human brain organoid, as siRNA-mediated knockdown of either PDGFRα or EGFR completely abrogated the susceptibility of brain organoids to TB40/E. Unlike previous findings with fibroblasts and epithelial cells that implicate either PDGFRα or EGFR as important receptor for HCMV entry into host cells, our brain organoid study suggests that both PDGFRα and EGFR are important for efficient HCMV infection of cell types in the developing human brain. This observation suggests that infection of human brain cells by HCMV could be orchestrated by multiple HCMV envelope glycoprotein complexes.

By taking advantage of two previously isolated NAbs, 1B2 and 62-11,[36,37] that were raised against the HCMV PC by vaccination, we have shown that NAbs targeting epitopes of the PC can potently prevent the infection of brain organoids by HCMV, thereby allowing normal human brain organoid development. HCMV NAbs targeting the PC or other envelope glycoprotein complexes has been characterized using various cell types, including fibroblasts, epithelial/endothelial cells, monocytes/macrophages, and cells of the human placenta.[36,56-58] Yet the capacity of NAbs to prevent HCMV infection of human brain cells has not been reported. This study demonstrates that both 1B2 and 62-11 NAbs can potently prevent HCMV infection of human brain organoids, suggesting that NAbs targeting the PC may have the capacity to effectively prevent HCMV infection of the developing human brain. Notably, the maximum NAb concentration (4 μg/mL) used to model the prevention of HCMV infection of the brain organoids was more than 50 times lower than monoclonal antibody concentrations found in human serum post-intravenous infusion of PC-specific NAbs in healthy adults or kidney transplant recipients.[59,60] Considering that fetal IgG concentrations at weeks 17–22 are about 5%–10% of the maternal IgG levels,[61] this may suggest that maternal monoclonal antibody concentrations of 20–40 μg/mL could potentially reach the fetal compartment at a time point of gestation when HCMV-induced fetal brain damage is most severe.[7] As the embryo blood-brain-barrier is considered to be immature or “leaky,” antibodies that enter the fetal circulation from the mother during early gestation are thought to have direct access to the fetal brain,[62,63] suggesting that vaccine-induced or passively administered NAbs may have the potential to prevent HCMV-related brain diseases during early fetal development. Given that the antibodies used in this study were isolated from PC-immunized mice, clinical application of these antibodies to reduce or prevent neurodevelopmental defects caused by HCMV would require their humanization to allow for Fc binding and to prevent development of a potentially harmful human anti-mouse antibody response.[64]

There has been controversy about the protective capacity of antibodies in prevention of congenital HCMV infection,[32,65] especially in light of imperfect protection by naturally acquired HCMV immunity.[3] Although a recent study using samples from a high seroprevalance population did not find a correlation between PC-specific or gH/gL/gO-specific antibody titers and the prevention of congenital transmission,[66] other studies reported a correlation between PC-specific or gB-specific antibodies and reduced intrauterine HCMV transmission following primary maternal infection.[33-35] In addition, a recent study using the highly relevant rhesus macaque model of congenital CMV infection has shown that passively transferred, pre-existing antibodies with high neutralizing activity can protect against CMV vertical transmission and fetal demise.[67] Although the precise reasons for the difference in the protective capacity of CMV-specific antibodies observed in these studies are unclear, they could be related to differences in epitope specificity, affinity, potency, or function of the antibody response. Our study implies that pre-existing PC-specific or gH-specific NAbs transferred from the mother to the fetus following passive or active immunization may have the capacity to prevent severe fetal brain malformation by HCMV that has crossed the fetal-maternal interface.

In summary, we have used hiPSC-derived brain organoids to model the effects of HCMV infection on early human brain development with different HCMV stains. We have shown that HCMV infection of hiPSC-derived brain organoids can result in severely impaired brain organoid growth and structure as well as altered calcium signaling and neural network activity, phenotypes that mimic HCMV-induced neurological conditions, such as microcephaly. In addition, we have shown that severely impaired brain organoid development caused by HCMV can be effectively prevented by NAbs that target the HCMV PC, suggesting that PC-specific NAbs may have the capacity to prevent HCMV-induced neurodevelopmental deficits. This 3D organoid model provides a powerful system to study HCMV-induced brain malformation and to identify potential antiviral agents that could prevent abnormal brain development due to congenital HCMV infection.

STAR★METHODS

Detailed methods are provided in the online version of this paper and include the following:

- KEY RESOURCES TABLE
- LEAD CONTACT AND MATERIALS AVAILABILITY
- EXPERIMENTAL MODEL AND SUBJECT DETAILS
  - Human iPSC Derivation
  - Cell Lines
  - Viruses
- METHOD DETAILS
  - Generation of brain organoids from hiPSCs
  - HCMV infection of brain organoids
  - Brain organoid treatment with NAbs
  - Immunostaining
  - Calcium imaging
  - Microelectrode Arrays (MEA)
  - Generation of NPCs from human iPSCs
  - Neuronal differentiation from NPCs
  - RNA-sequencing

CellPress
OPEN ACCESS

- RT-qPCR analysis
- RNA interference
- Western blot analysis
- Plasmids
- hiPSC electroporation and HCMV infection
- QUANTIFICATION AND STATISTICAL ANALYSIS
  - Statistical analysis
- DATA AND CODE AVAILABILITY

SUPPLEMENTAL INFORMATION

Supplemental Information can be found online at https://doi.org/10.1016/j.xcrm.2020.100002.

ACKNOWLEDGMENTS

We thank Louise and Herbert Horvitz for their generosity and forethought; Dr. John Zaia for advice on CMV-related topics; Drs. Xiwei Wu, Jinhui Wang, and Juan Du for help with RNA-seq analysis and data deposit; Dr. Lizhao Feng for providing IMR 90 NPCs; Dr. Guihua Sun for generating the pCDNA-PDGFRα plasmid; Mr. Michael Nelson and Dr. Brian Armstrong in Imaging Core Facility of City of Hope for imagining assistance; and Dr. Xiuli Wang for providing the pHIV-T2A-DsRed plasmid. G.S. was a Herbert Horvitz Fellow. This work was supported by the Louise and Herbert Horvitz Charitable Foundation; Sidell Kagan Foundation; California Institute for Regenerative Medicine TRAN1-08525; and the National Institute of Aging of the National Institutes of Health R01AG056305, RF1AG061794, and R56AG061171 to Y.S., the National Institutes of Health U19AI131130 and R35NS097370 to G.-L.M., R37NS047344 to H.S., in part by the Leona M. and Harry B. Helmsley Charitable Trust no. 2017-PG-MED001; Grace Foundation; JPB Foundation; Annette C. Merle-Smith; Robert and Mary Jane Engman Foundation; Lynn and Edward Streim; and the Ray and Dagmar Dolby Family Fund to F.H.G., and in part by US Public Health Service grant R01 AI103960 to D.J.D., who was partially supported by R01CA077544, R01CA181045, P01CA111412, P50CA107399, R01HD079918, and U19AI128913. The City of Hope Cancer Center is supported by the National Cancer Institute of the National Institutes of Health under award number CA33572. Research reported in this publication included work performed in the Analytical Cytometry and Integrative Genomics Cores supported by the National Cancer Institute of the National Institutes of Health under award number P30CA33572. The content of this work is solely the responsibility of the authors and does not necessarily represent the official views of the National Institutes of Health.

AUTHOR CONTRIBUTIONS

Y.S. conceived the project. G.S. and X.C. generated brain organoids. X.C. and G.S. designed and performed Ca imaging assay. G.S. designed, performed, and analyzed the brain organoid MEA experiments. G.S., F.W., and F.C. designed and performed receptor experiments. G.S., F.W., and F.C. designed and performed brain organoid microcephaly experiments. F.W., F.C., and G.S. designed NAb experiments. G.S. performed NAb experiments. F.W. and F.C. provided HCMV stocks and validated the virus in different cell types. C.W. performed neuronal differentiation from hiPSC-derived NPCs. E.T. generated AG14048 iPSCs. J.N. and M.K. helped to produce HCMV stocks. J.N. prepared the EGFR-expression vector. G.S., D.T., H.Z., J.N., and Y.S. analyzed data. G.S. and F.W. wrote the first draft of the manuscript. Y.S., F.W., F.C., and G.S. edited and finalized the manuscript with comments from all other authors. M.C.M. and F.H.G. helped with MEA setup. H.S. and G.-L.M. helped with brain organoid setup. Y.S. and D.J.D. provided senior supervision of the study.

DECLARATION OF INTERESTS

F.W., F.C., and D.J.D. receive royalty payments from Helocyte Inc. D.J.D. chairs the Scientific Advisory Board of Helocyte Inc., and he has an equity interest in Helocyte Inc. F.W., F.C., and D.J.D. hold patents US10487139B2 and US10376575B2.

Received: May 2, 2019
Revised: January 16, 2020
Accepted: February 28, 2020
Published: March 25, 2020

REFERENCES

1. Britt, W. (2008). Manifestations of human cytomegalovirus infection: proposed mechanisms of acute and chronic disease. Curr. Top. Microbiol. Immunol. *325*, 417–470.
2. Cheeran, M.C., Lokensgard, J.R., and Schleiss, M.R. (2009). Neuropathogenesis of congenital cytomegalovirus infection: disease mechanisms and prospects for intervention. Clin. Microbiol. Rev. *22*, 99–126.
3. Britt, W.J. (2017). Congenital human cytomegalovirus infection and the enigma of maternal immunity. J. Virol. *91*, e02392-16.
4. Boppana, S.B., Rivera, L.B., Fowler, K.B., Mach, M., and Britt, W.J. (2001). Intrauterine transmission of cytomegalovirus to infants of women with preconceptional immunity. N. Engl. J. Med. *344*, 1366–1371.
5. Kenneson, A., and Cannon, M.J. (2007). Review and meta-analysis of the epidemiology of congenital cytomegalovirus (CMV) infection. Rev. Med. Virol. *17*, 253–276.
6. Lanzieri, T.M., Dollard, S.C., Bialek, S.R., and Grosse, S.D. (2014). Systematic review of the birth prevalence of congenital cytomegalovirus infection in developing countries. Int. J. Infect. Dis. *22*, 44–48.
7. Wang, C., Zhang, X., Bialek, S., and Cannon, M.J. (2011). Attribution of congenital cytomegalovirus infection to primary versus non-primary maternal infection. Clin. Infect. Dis. *52*, e11–e13.
8. Teissier, N., Fallet-Bianco, C., Delezoide, A.L., Laquerrière, A., Marcorelles, P., Khung-Savatovsky, S., Nardelli, J., Cipriani, S., Csaba, Z., Picone, O., et al. (2014). Cytomegalovirus-induced brain malformations in fetuses. J. Neuropathol. Exp. Neurol. *73*, 143–158.
9. Cannon, M.J., and Davis, K.F. (2005). Washing our hands of the congenital cytomegalovirus disease epidemic. BMC Public Health *5*, 70.
10. Kawasaki, H., Kosugi, I., Meguro, S., and Iwashita, T. (2017). Pathogenesis of developmental anomalies of the central nervous system induced by congenital cytomegalovirus infection. Pathol. Int. *67*, 72–82.
11. Taverna, E., Götz, M., and Huttner, W.B. (2014). The cell biology of neurogenesis: toward an understanding of the development and evolution of the neocortex. Annu. Rev. Cell Dev. Biol. *30*, 465–502.
12. Di Lullo, E., and Kriegstein, A.R. (2017). The use of brain organoids to investigate neural development and disease. Nat. Rev. Neurosci. *18*, 573–584.
13. Lancaster, M.A., and Knoblich, J.A. (2014). Organogenesis in a dish: modeling development and disease using organoid technologies. Science *345*, 1247125.
14. Qian, X., Nguyen, H.N., Song, M.M., Hadiono, C., Ogden, S.C., Hammack, C., Yao, B., Hamersky, G.R., Jacob, F., Zhong, C., et al. (2016). Brain-region-specific organoids using mini-bioreactors for modeling ZIKV exposure. Cell *165*, 1238–1254.
15. Lancaster, M.A., and Knoblich, J.A. (2014). Generation of cerebral organoids from human pluripotent stem cells. Nat. Protoc. *9*, 2329–2340.
16. Petersen, L.R., Jamieson, D.J., Powers, A.M., and Honein, M.A. (2016). Zika virus. N. Engl. J. Med. *374*, 1552–1563.
17. Cugola, F.R., Fernandes, I.R., Russo, F.B., Freitas, B.C., Dias, J.L., Guimarães, K.P., Benazzato, C., Almeida, N., Pignatari, G.C., Romero, S., et al. (2016). The Brazilian Zika virus strain causes birth defects in experimental models. Nature *534*, 267–271.
18. Lancaster, M.A., Renner, M., Martin, C.A., Wenzel, D., Bicknell, L.S., Hurles, M.E., Homfray, T., Penninger, J.M., Jackson, A.P., and Knoblich,

J.A. (2013). Cerebral organoids model human brain development and microcephaly. Nature *501*, 373–379.

19. Kelava, I., and Lancaster, M.A. (2016). Dishing out mini-brains: current progress and future prospects in brain organoid research. Dev. Biol. *420*, 199–209.

20. Li, W., Sun, G., Yang, S., Qu, Q., Nakashima, K., and Shi, Y. (2008). Nuclear receptor TLX regulates cell cycle progression in neural stem cells of the developing brain. Mol. Endocrinol. *22*, 56–64.

21. Mora-Bermúdez, F., Badsha, F., Kanton, S., Camp, J.G., Vernot, B., Köhler, K., Voigt, B., Okita, K., Maricic, T., He, Z., et al. (2016). Differences and similarities between human and chimpanzee neural progenitors during cerebral cortex development. eLife *5*, e18683.

22. Turrigiano, G.G., Leslie, K.R., Desai, N.S., Rutherford, L.C., and Nelson, S.B. (1998). Activity-dependent scaling of quantal amplitude in neocortical neurons. Nature *391*, 892–896.

23. Molyneaux, B.J., Arlotta, P., Menezes, J.R., and Macklis, J.D. (2007). Neuronal subtype specification in the cerebral cortex. Nat. Rev. Neurosci. *8*, 427–437.

24. Gaspard, N., Bouschet, T., Hourez, R., Dimidschstein, J., Naeije, G., van den Ameele, J., Espuny-Camacho, I., Herpoel, A., Passante, L., Schiffmann, S.N., et al. (2008). An intrinsic mechanism of corticogenesis from embryonic stem cells. Nature *455*, 351–357.

25. O'Connor, C.M., and Murphy, E.A. (2012). A myeloid progenitor cell line capable of supporting human cytomegalovirus latency and reactivation, resulting in infectious progeny. J. Virol. *86*, 9854–9865.

26. Cherrington, J.M., and Mocarski, E.S. (1989). Human cytomegalovirus ie1 transactivates the alpha promoter-enhancer via an 18-base-pair repeat element. J. Virol. *63*, 1435–1440.

27. Hahn, G., Revello, M.G., Patrone, M., Percivalle, E., Campanini, G., Sarasini, A., Wagner, M., Gallina, A., Milanesi, G., Koszinowski, U., et al. (2004). Human cytomegalovirus UL131-128 genes are indispensable for virus growth in endothelial cells and virus transfer to leukocytes. J. Virol. *78*, 10023–10033.

28. Wang, D., and Shenk, T. (2005). Human cytomegalovirus virion protein complex required for epithelial and endothelial cell tropism. Proc. Natl. Acad. Sci. USA *102*, 18153–18158.

29. Ryckman, B.J., Rainish, B.L., Chase, M.C., Borton, J.A., Nelson, J.A., Jarvis, M.A., and Johnson, D.C. (2008). Characterization of the human cytomegalovirus gH/gL/UL128-131 complex that mediates entry into epithelial and endothelial cells. J. Virol. *82*, 60–70.

30. Wussow, F., Chiuppesi, F., Contreras, H., and Diamond, D.J. (2017). Neutralization of human cytomegalovirus entry into fibroblasts and epithelial cells. Vaccines (Basel) *5*, E39.

31. Falk, J.J., Laib Sampaio, K., Stegmann, C., Lieber, D., Kropff, B., Mach, M., and Sinzger, C. (2016). Generation of a Gaussia luciferase-expressing endotheliotropic cytomegalovirus for screening approaches and mutant analyses. J. Virol. Methods *235*, 182–189.

32. Nigro, G., Adler, S.P., La Torre, R., and Best, A.M.; Congenital Cytomegalovirus Collaborating Group (2005). Passive immunization during pregnancy for congenital cytomegalovirus infection. N. Engl. J. Med. *353*, 1350–1362.

33. Lilleri, D., Kabanova, A., Lanzavecchia, A., and Gerna, G. (2012). Antibodies against neutralization epitopes of human cytomegalovirus gH/gL/pUL128-130-131 complex and virus spreading may correlate with virus control in vivo. J. Clin. Immunol. *32*, 1324–1331.

34. Lilleri, D., Kabanova, A., Revello, M.G., Percivalle, E., Sarasini, A., Genini, E., Sallusto, F., Lanzavecchia, A., Corti, D., and Gerna, G. (2013). Fetal human cytomegalovirus transmission correlates with delayed maternal antibodies to gH/gL/pUL128-130-131 complex during primary infection. PLoS ONE *8*, e59863.

35. Boppana, S.B., and Britt, W.J. (1995). Antiviral antibody responses and intrauterine transmission after primary maternal cytomegalovirus infection. J. Infect. Dis. *171*, 1115–1121.

36. Chiuppesi, F., Wussow, F., Johnson, E., Bian, C., Zhuo, M., Rajakumar, A., Barry, P.A., Britt, W.J., Chakraborty, R., and Diamond, D.J. (2015). Vaccine-derived neutralizing antibodies to the human cytomegalovirus gH/gL pentamer potently block primary cytotrophoblast infection. J. Virol. *89*, 11884–11898.

37. Wussow, F., Chiuppesi, F., Martinez, J., Campo, J., Johnson, E., Flechsig, C., Newell, M., Tran, E., Ortiz, J., La Rosa, C., et al. (2014). Human cytomegalovirus vaccine based on the envelope gH/gL pentamer complex. PLoS Pathog. *10*, e1004524.

38. Murphy, E., Yu, D., Grimwood, J., Schmutz, J., Dickson, M., Jarvis, M.A., Hahn, G., Nelson, J.A., Myers, R.M., and Shenk, T.E. (2003). Coding potential of laboratory and clinical strains of human cytomegalovirus. Proc. Natl. Acad. Sci. USA *100*, 14976–14981.

39. Soroceanu, L., Akhavan, A., and Cobbs, C.S. (2008). Platelet-derived growth factor-alpha receptor activation is required for human cytomegalovirus infection. Nature *455*, 391–395.

40. Wang, X., Huong, S.M., Chiu, M.L., Raab-Traub, N., and Huang, E.S. (2003). Epidermal growth factor receptor is a cellular receptor for human cytomegalovirus. Nature *424*, 456–461.

41. Feire, A.L., Koss, H., and Compton, T. (2004). Cellular integrins function as entry receptors for human cytomegalovirus via a highly conserved disintegrin-like domain. Proc. Natl. Acad. Sci. USA *101*, 15470–15475.

42. Kabanova, A., Marcandalli, J., Zhou, T., Bianchi, S., Baxa, U., Tsybovsky, Y., Lilleri, D., Silacci-Fregni, C., Foglierini, M., Fernandez-Rodriguez, B.M., et al. (2016). Platelet-derived growth factor-α receptor is the cellular receptor for human cytomegalovirus gHgLgO trimer. Nat. Microbiol. *1*, 16082.

43. D'Aiuto, L., Di Maio, R., Heath, B., Raimondi, G., Milosevic, J., Watson, A.M., Bamne, M., Parks, W.T., Yang, L., Lin, B., et al. (2012). Human induced pluripotent stem cell-derived models to investigate human cytomegalovirus infection in neural cells. PLoS ONE *7*, e49700.

44. Qin, J., Chai, G., Brewer, J.M., Lovelace, L.L., and Lebioda, L. (2006). Fluoride inhibition of enolase: crystal structure and thermodynamics. Biochemistry *45*, 793–800.

45. Chaanine, A.H., Gordon, R.E., Kohlbrenner, E., Benard, L., Jeong, D., and Hajjar, R.J. (2013). Potential role of BNIP3 in cardiac remodeling, myocardial stiffness, and endoplasmic reticulum: mitochondrial calcium homeostasis in diastolic and systolic heart failure. Circ. Heart Fail. *6*, 572–583.

46. Shumilina, E., Zemtsova, I.M., Heise, N., Schmid, E., Eichenmüller, M., Tyan, L., Rexhepaj, R., and Lang, F. (2010). Phosphoinositide-dependent kinase PDK1 in the regulation of Ca2+ entry into mast cells. Cell. Physiol. Biochem. *26*, 699–706.

47. Gerna, G., Revello, M.G., Baldanti, F., Percivalle, E., and Lilleri, D. (2017). The pentameric complex of human cytomegalovirus: cell tropism, virus dissemination, immune response and vaccine development. J. Gen. Virol. *98*, 2215–2234.

48. Brown, R.M., Rana, P.S.J.B., Jaeger, H.K., O'Dowd, J.M., Balemba, O.B., and Fortunato, E.A. (2019). Human cytomegalovirus compromises development of cerebral organoids. J. Virol. *93*, e00957-19.

49. Sison, S.L., O'Brien, B.S., Johnson, A.J., Seminary, E.R., Terhune, S.S., and Ebert, A.D. (2019). Human cytomegalovirus disruption of calcium signaling in neural progenitor cells and organoids. J. Virol. *93*, e00954-19.

50. Manicklal, S., Emery, V.C., Lazzarotto, T., Boppana, S.B., and Gupta, R.K. (2013). The "silent" global burden of congenital cytomegalovirus. Clin. Microbiol. Rev. *26*, 86–102.

51. Kwak, M., Yum, M.S., Yeh, H.R., Kim, H.J., and Ko, T.S. (2018). Brain magnetic resonance imaging findings of congenital cytomegalovirus infection as a prognostic factor for neurological outcome. Pediatr. Neurol. *83*, 14–18.

52. Ciferri, C., Chandramouli, S., Donnarumma, D., Nikitin, P.A., Cianfrocco, M.A., Gerrein, R., Feire, A.L., Barnett, S.W., Lilja, A.E., Rappuoli, R., et al. (2015). Structural and biochemical studies of HCMV gH/gL/gO and Pentamer reveal mutually exclusive cell entry complexes. Proc. Natl. Acad. Sci. USA *112*, 1767–1772.

CellPress
OPEN ACCESS

53. Wu, Y., Prager, A., Boos, S., Resch, M., Brizic, I., Mach, M., Wildner, S., Scrivano, L., and Adler, B. (2017). Human cytomegalovirus glycoprotein complex gH/gL/gO uses PDGFR-α as a key for entry. PLoS Pathog. *13*, e1006281.
54. Martinez-Martin, N., Marcandalli, J., Huang, C.S., Arthur, C.P., Perotti, M., Foglierini, M., Ho, H., Dosey, A.M., Shriver, S., Payandeh, J., et al. (2018). An unbiased screen for human cytomegalovirus identifies neuropilin-2 as a central viral receptor. Cell *174*, 1158–1171.e19.
55. Ryckman, B.J., Chase, M.C., and Johnson, D.C. (2008). HCMV gH/gL/UL128-131 interferes with virus entry into epithelial cells: evidence for cell type-specific receptors. Proc. Natl. Acad. Sci. USA *105*, 14118–14123.
56. Kabanova, A., Perez, L., Lilleri, D., Marcandalli, J., Agatic, G., Becattini, S., Preite, S., Fuschillo, D., Percivalle, E., Sallusto, F., et al. (2014). Antibody-driven design of a human cytomegalovirus gHgLpUL128L subunit vaccine that selectively elicits potent neutralizing antibodies. Proc. Natl. Acad. Sci. USA *111*, 17965–17970.
57. Zydek, M., Petitt, M., Fang-Hoover, J., Adler, B., Kauvar, L.M., Pereira, L., and Tabata, T. (2014). HCMV infection of human trophoblast progenitor cells of the placenta is neutralized by a human monoclonal antibody to glycoprotein B and not by antibodies to the pentamer complex. Viruses *6*, 1346–1364.
58. Macagno, A., Bernasconi, N.L., Vanzetta, F., Dander, E., Sarasini, A., Revello, M.G., Gerna, G., Sallusto, F., and Lanzavecchia, A. (2010). Isolation of human monoclonal antibodies that potently neutralize human cytomegalovirus infection by targeting different epitopes on the gH/gL/UL128-131A complex. J. Virol. *84*, 1005–1013.
59. Deng, R., Wang, Y., Maia, M., Burgess, T., McBride, J.M., Liao, X.C., Tavel, J.A., and Hanley, W.D. (2018). Pharmacokinetics and exposure-response analysis of RG7667, a combination of two anticytomegalovirus monoclonal antibodies, in a phase 2a randomized trial to prevent cytomegalovirus infection in high-risk kidney transplant recipients. Antimicrob. Agents Chemother. *62*, e01108-17.
60. Ishida, J.H., Burgess, T., Derby, M.A., Brown, P.A., Maia, M., Deng, R., Emu, B., Feierbach, B., Fouts, A.E., Liao, X.C., and Tavel, J.A. (2015). Phase 1 randomized, double-blind, placebo-controlled study of RG7667, an anticytomegalovirus combination monoclonal antibody therapy, in healthy adults. Antimicrob. Agents Chemother. *59*, 4919–4929.
61. Palmeira, P., Quinello, C., Silveira-Lessa, A.L., Zago, C.A., and Carneiro-Sampaio, M. (2012). IgG placental transfer in healthy and pathological pregnancies. Clin. Dev. Immunol. *2012*, 985646.
62. Fox, E., Amaral, D., and Van de Water, J. (2012). Maternal and fetal antibrain antibodies in development and disease. Dev. Neurobiol. *72*, 1327–1334.
63. Saunders, N.R., Liddelow, S.A., and Dziegielewska, K.M. (2012). Barrier mechanisms in the developing brain. Front. Pharmacol. *3*, 46.
64. Maidji, E., McDonagh, S., Genbacev, O., Tabata, T., and Pereira, L. (2006). Maternal antibodies enhance or prevent cytomegalovirus infection in the placenta by neonatal Fc receptor-mediated transcytosis. Am. J. Pathol. *168*, 1210–1226.
65. Revello, M.G., Lazzarotto, T., Guerra, B., Spinillo, A., Ferrazzi, E., Kustermann, A., Guaschino, S., Vergani, P., Todros, T., Frusca, T., et al.; CHIP Study Group (2014). A randomized trial of hyperimmune globulin to prevent congenital cytomegalovirus. N. Engl. J. Med. *370*, 1316–1326.
66. Vanarsdall, A.L., Chin, A.L., Liu, J., Jardetzky, T.S., Mudd, J.O., Orloff, S.L., Streblow, D., Mussi-Pinhata, M.M., Yamamoto, A.Y., Duarte, G., et al. (2019). HCMV trimer- and pentamer-specific antibodies synergize for virus neutralization but do not correlate with congenital transmission. Proc. Natl. Acad. Sci. USA *116*, 3728–3733.
67. Nelson, C.S., Cruz, D.V., Tran, D., Bialas, K.M., Stamper, L., Wu, H., Gilbert, M., Blair, R., Alvarez, X., Itell, H., et al. (2017). Preexisting antibodies can protect against congenital cytomegalovirus infection in monkeys. JCI Insight *2*, 94002.
68. Andreoni, M., Faircloth, M., Vugler, L., and Britt, W.J. (1989). A rapid microneutralization assay for the measurement of neutralizing antibody reactive with human cytomegalovirus. J. Virol. Methods *23*, 157–167.
69. Greulich, H., Chen, T.H., Feng, W., Jänne, P.A., Alvarez, J.V., Zappaterra, M., Bulmer, S.E., Frank, D.A., Hahn, W.C., Sellers, W.R., and Meyerson, M. (2005). Oncogenic transformation by inhibitor-sensitive and -resistant EGFR mutants. PLoS Med. *2*, e313.
70. Johannessen, C.M., Boehm, J.S., Kim, S.Y., Thomas, S.R., Wardwell, L., Johnson, L.A., Emery, C.M., Stransky, N., Cogdill, A.P., Barretina, J., et al. (2010). COT drives resistance to RAF inhibition through MAP kinase pathway reactivation. Nature *468*, 968–972.
71. Niwa, H., Yamamura, K., and Miyazaki, J. (1991). Efficient selection for high-expression transfectants with a novel eukaryotic vector. Gene *108*, 193–199.
72. Kime, C., Rand, T.A., Ivey, K.N., Srivastava, D., Yamanaka, S., and Tomoda, K. (2015). Practical integration-free episomal methods for generating human induced pluripotent stem cells. Curr. Protoc. Hum. Genet. *87*, 21.2.1–21.2.21.
73. Li, L., Tian, E., Chen, X., Chao, J., Klein, J., Qu, Q., Sun, G., Sun, G., Huang, Y., Warden, C.D., et al. (2018). GFAP mutations in astrocytes impair oligodendrocyte progenitor proliferation and myelination in an hiPSC model of Alexander disease. Cell Stem Cell *23*, 239–251.e6.
74. Britt, W.J. (2010). Human cytomegalovirus: propagation, quantification, and storage. Curr. Protoc. Microbiol. *Chapter 14*, Unit 14E.3.
75. Douvaras, P., and Fossati, V. (2015). Generation and isolation of oligodendrocyte progenitor cells from human pluripotent stem cells. Nat. Protoc. *10*, 1143–1154.
76. Kim, D., Pertea, G., Trapnell, C., Pimentel, H., Kelley, R., and Salzberg, S.L. (2013). TopHat2: accurate alignment of transcriptomes in the presence of insertions, deletions and gene fusions. Genome Biol. *14*, R36.

STAR★METHODS

KEY RESOURCES TABLE

REAGENT or RESOURCE	SOURCE	IDENTIFIER
Antibodies		
Mouse monoclonal anti-CMV IE-1 clone p63-27	Andreoni et al.[68]	N/A
Mouse monoclonal anti-CMV PC clone 1B2	Chiuppesi et al.[36]	N/A
Mouse monoclonal anti-CMV gH clone 62-11	Chiuppesi et al.[36]	N/A
Normal mouse IgG	Santa Cruz Biotechnologies	Cat# sc-2025; RRID:AB_737182
Rabbit anti-TUJ1	Covance	Cat# PRB-435P; RRID:AB_10616742
Chicken anti-MAP2	Abcam	Cat# Abcam ab5392; RRID:AB_2138153
Goat anti-SOX2	Santa Cruz Biotechnologies	Cat# sc-17320; RRID:AB_2286684
Rat anti-CTIP2	Abcam	Cat# ab18465; RRID:AB_2064130
Mouse anti-SABT2	Abcam	Cat# ab92446; RRID:AB_10563678
Rabbit anti-TRB2	Abcam	Cat# ab31940; RRID:AB_2200219
Rabbit anti-BRN2	Santa Cruz Biotechnologies	Cat# sc-28594 RRID:AB_2167382
Rabbit anti-TLX	Li et al.[20]	N/A
mAb Rat anti-BrdU	Accurate Chemical & Scientific Corp	Cat# OBT0030 RRID:AB_2341179
Rabbit anti-cleaved Caspase-3	Cell Signaling	Cat# 9661 RRID:AB_2341188
Bacterial and Virus Strains		
TB40/E-GFP	O'Connor and Murphy[25]	N/A
TR-GFP	Murphy et al.[38]	N/A
TOWNE-GFP (clone RC2940)	Cherrington and Mocarski[26]	N/A
TB40/E-Gluc	Falk et al.[31]	N/A
Chemicals, Peptides, and Recombinant Proteins		
ROCK inhibitor Y-27632 dihydrochloride	Stemgent / Reprocell	Cat # 04-0012-10
Bicuculline	Tocris	Cat # 0130
Retinoic acid	Sigma	Cat # R2625
CHIR99021	Cellagen Technology	Cat # C2447-2 s
LDN-193189	Cellagen Technology	Cat # C5361-2 s
SB431542	Cellagen Technology	Cat # C7243-5
Matrigel	Corning	Cat # 354230
CNQX disodium salt	Tocris	Cat # 1045
N2 supplement	Life Technologies	Cat # 17502048
B27 *supplement*	Thermo Fisher Scientific	Cat # 17504044
Essential 8 Medium	Life Technologies	Cat # A1517001
Human Recombinant Insulin solution	Sigma	Cat # I9278
MEM NEAA	Thermo Fisher Scientific	Cat # 11140076
DMEM-F12	GIBCO	Cat # 11330-032
BrainPhys medium	STEMCELL Tech	Cat # 05790
GDNF	PeproTech	Cat # 450-10
BDNF	PeproTech	Cat # 450-02
Dibutyryl-cAMP	Sigma Aldrich	Cat # D0627
ATRA	Sigma Aldrich	Cat # R2625
EGF	PeproTech	Cat # 100-15
FGF	PeproTech	Cat # 100-18B

(*Continued on next page*)

CellPress OPEN ACCESS

Continued

REAGENT or RESOURCE	SOURCE	IDENTIFIER
Trizol	Invitrogen	Cat # 15596018
GIBCO GlutaMAX Supplement	Invitrogen	Cat # 35050079
siLentFect Lipid Reagent for RNAi	Bio-Rad	Cat # 1703361
DyNAmo Flash SYBR Green qPCR mix	Thermo Fisher Scientific	Cat # F416
PMSF	Roche	Cat # 837091
Fluo-4, AM	Thermo Fisher Scientific	*Cat # F14201*
Critical Commercial Assays		
VECTASTAIN Elite ABC HRP Kit (Peroxidase, Mouse IgG)	Vector Laboratories	Cat # PK-6102
DAB Peroxidase (HRP) Substrate Kit, 3,3′-diaminobenzidine	Vector Laboratories	Cat # SK-4100
Tetro cDNA synthesis kit	Bioline	Cat # Bio-65043
Amaxa P3 primary 4D-Nucleofector X kit L	Lonza	Cat # V4XP-3024
Deposited Data		
RNaseq data of mock and hCMV infected brain organoids	This study	GSE145415
Experimental Models: Cell Lines		
Human: IMR-90 fibroblasts	Coriell Institute	Cat # I90-10
Human: AG14048 fibroblasts	Coriell Institute	Cat # AG14048
Human: ARPE-19	ATCC	Cat # CRL-2302
Human: MRC-5	ATCC	Cat # CCL-171
Oligonucleotides		
EGFR-siRNA: 5′-GAC GGC GUC CGC AAG UGU A −3′ (sense) and 5′-UAC ACU UGC GGA CGC CGU CUU-3′ (antisense)	This study	N/A
PDGFRα-specific siRNA: 5′-CCA CCU UCA UCA AGA GAG A-3′(sense) and 5′-UCU CUC UUG AUG AAG GUG GAA-3′ (antisense)	This study	N/A
ITGA3-specific siRNA: 5′-GCU ACA UGA UUC AGC GCA A-3′ (sense) and 5′-UUG CGC UGA AUC AUG UAG CUG-3′ (antisense)	This study	N/A
ITGA5-specific siRNA: 5′-GGG AAC CUC ACU UAC GGC U-3′ (sense) and 5′-AGC CGU AAG UGA GGU UCC CUU-3′ (antisense)	Dharmacon	Cat# L-004124-00-0005
Integrin β3 siRNA: 5′-CCA GAU GCC UGC ACC UUU A-3′, 5′-GCC AAC AAC CCA CUG UAU A-3′, 5′-GAA GAA CGC GCC AGA GCA A-3′, and 5′-GCA GUG AAU UGU ACC UAU A-3′	This study	N/A
Recombinant DNA		
pHIV-T2A-DsRed plasmid	Dr. Xiuli Wang from City of Hope	N/A
pBABE-EGFR WT plasmid	Greulich et al.[69]	Addgene Cat# 11011; RRID:Addgene_11011
pDONR223-PDGFRA plasmid	Johannessen et al.[70]	Addgene Cat# 23892; RRID:Addgene_23892
pCXLE-hSK	Addgene	Cat# 27078
pCXLE-hUL	Addgene	Cat# 27080
pCXLE-hOCT3/4-shp53-F	Addgene	Cat# 27077
pCXWB-EBNA1	Addgene	Cat# 37624

(*Continued on next page*)

Continued

REAGENT or RESOURCE	SOURCE	IDENTIFIER
pcDNA-PDGFRα	This study	N/A
pHIV-EGFR-T2A-DsRed	This study	N/A
Software and Algorithms		
Prism version 7	Graphpad Prism	https://www.graphpad.com/scientific-software/prism/
ZEN software	Carl Zeiss	https://www.zeiss.com/microscopy/us/products/microscope-software/zen.html
Image-Pro Premier 9.1	Media Cybernetics	https://www.mediacy.com/support/imagepropremier
AxIS software	Axion Biosystems	https://www.axionbiosystems.com/products/axis-software
AxIS Metrics Tool	Axion Biosystems	https://www.axionbiosystems.com/products/axis-software
NeuralMetric Tool	Axion Biosystems	https://www.axionbiosystems.com/products/axis-software
ClustVis	N/A	https://biit.cs.ut.ee/clustvis/
Other		
CytoView MEA 12 plate	Axion BioSystems	Cat # M768-GL1-30Pt200-5
Maestro MEA system	Axion BioSystems	Equipment
Hamamatsu EMCCD	Hamamatsu	Model C9100-13
Orbi-Shaker	Benchmark Scientific	NC0483060

LEAD CONTACT AND MATERIALS AVAILABILITY

Further information and requests for resources and reagents should be directed to and will be fulfilled by the Lead Contact, Yanhong Shi (yshi@coh.org). This study did not generate new unique reagents.

EXPERIMENTAL MODEL AND SUBJECT DETAILS

Human iPSC Derivation

AG14048 and IMR90 (I90) human fibroblasts were purchased from Coriell. AG14048 iPSCs were generated from AG14048 fibroblasts through episomal reprogramming using episomal plasmids expressing OCT4, SOX2, L-MYC, KLF4, shp53, and EBNA1 (Addgene plasmids pCXLE-hSK, pCXLE-hUL, pCXLE-hOCT3/4-shp53-F, and pCXWB-EBNA1)[71,72]. Derivation of I90 iPSCs were described previously[73]. Specifically, human fibroblast cells were electroporated with the reprogramming factors using 4D Nucleofector (Lonza) and seeded into 6-well plates coated with 1:100 diluted Matrigel (Corning) and maintained in E8 medium (Invitrogen). iPSCs were maintained at 37°C in Matrigel-coated 6-well plates with daily medium change and passaged every 3-4 days using 0.5 mM EDTA (GIBCO) treatment and manual dissociation. Both AG14048 and I90 iPSC lines generated in this study were authenticated using short tandem repeat (STR) assay.

Cell Lines

ARPE-19 (male) and MRC-5 (male) cells were purchased from ATCC and maintained in Dulbecco's minimal essential medium (DMEM) or minimal essential medium (MEM), respectively, supplemented with 10% fetal bovine serum (FBS). All cell lines were cultured at 37°C and 5% CO_2.

Viruses

GFP-labeled TB40/E and TR viruses that express a GFP reporter under the SV40 promoter were derived from BAC DNA, kindly provided by T. E. Shenk and E. A. Murphy (Princeton University, NJ)[25,38]. GFP-tagged Towne virus (RC2940) that expresses an IE2/GFP fusion protein, was obtained from E. S. Mocarski (Emory University School of Medicine, GA)[26]. TB40/E-Gluc virus expressing a luciferase gene under the IE promoter was reconstituted from BAC DNA, kindly provided by S. Sinzger (Ulm University, Germany)[31]. TB40/E viral stocks were generated following viral propagation in ARPE-19 cells, whereas Towne virus stocks were generated following viral propagation in MRC-5. Viral stocks were generated by standard ultracentrifugation procedures as described previously[37,74] and titrated on MRC-5 by immunostaining using the IE1-specific monoclonal antibody p63-27[68] (kindly provided by William

Britt, University of Alabama at Birmingham) and the Vectastain ABC kit and 3,3′-diaminobenzidine (DAB) substrate according to the manufacturer's instructions. Images were taken using a DMi8 inverted microscope equipped with a linear motorized stage.

METHOD DETAILS

Generation of brain organoids from hiPSCs

hiPSC-derived brain organoids were generated based on the protocol described by Lancaster et al.[15] with modifications. Briefly, hiPSCs were generated from IMR-90 and AG14048 human fibroblasts, confirmed to be karyotypically normal and negative for mycoplasma contamination. On day 0 of organoid culture, hiPSCs were dissociated with EDTA, and seeded in suspension in a 6-well plate to form embryoid bodies in E8 medium with 5 μM ROCK inhibitor Y-27632. From day 1 to day 4, cells were cultured in E8 medium without ROCK inhibitor with daily medium change. On day 5, E8 medium was replaced by neural induction medium (NIM) containing DMEM-F12, 1 × N2 supplement, 1 × minimum essential medium NEAA (*MEM-NEAA*), and 2 μg/ml Heparin. On day 8, the spheres were embedded in 20%–25% Matrigel in NIM in a 6-well suspension plate and incubated at 37°C for 4 hr, followed by gentle addition of 2 mL of the NIM. On day 10-12, brain organoids were lifted and transferred to a new 6-well plate. NIM was changed daily from day 5 to day 15. On day 15, brain organoids were transferred to a T25 suspension culture flask and cultured in differentiation medium containing DMEM-F12, 1 × N2 supplement, 2.5 μg/ml Insulin, 1 × Glutamax, 0.5 × MEM-NEAA, 3.5 μl/L (V/V) 2-Mercaptoethanol, and 1 × B27 supplement on an Orbi-Shaker (Benchmark Scientific) at 50 rpm rotating speed. Medium was changed every 2-3 days. Organoids that exhibited similar size and passed the quality control criteria described by Lancaster et al.[15,18] were used for the study. The criteria include clear embryoid body border, formation of organized neuroepithelium before embedding, formation of ventricle-like structure, and development of defined bud in Matrigel without premature differentiation.

HCMV infection of brain organoids

Brain organoids at day 45 of differentiation were seeded in 24-well plates and exposed to 5×10^5 pfu/ml of HCMV virus for 24 hr. Following infection, 1 mL medium was replaced, and each organoid was placed in a single well for the duration of the experiment. At different time points post infection, each organoid was evaluated for GFP fluorescence intensity and organoid size in diameter using the same microscopy setting during each experiment.

Brain organoid treatment with NAbs

1B2 and 62-11 NAbs at final concentrations ranging from 3.2 ng/ml to 4000 ng/ml were incubated with 5×10^5 pfu/ml of HCMV for 1 hr at 37°C and then transferred to brain organoid-containing wells. Normal mouse IgG was used as a control at the concentration of 2000 ng/ml. For proliferation assay, brain organoids were incubated with 10 μM BrdU for 2 hr and fixed in 4% paraformaldehyde (PFA) for 1 hr, followed by immunostaining.

Immunostaining

Cells on coverslip were fixed with 4% PFA in PBS for 15 min at room temperature. Brain organoids were fixed with 4% PFA for 1 hr and submerged in 30% sucrose overnight. The samples were embedded in OCT and sectioned at a thickness of 14 or 20 μm using Leica CM3050S. Cells or brain organoid sections were permeabilized and blocked with blocking solution (1 × PBS containing 0.1% Triton X-100 and 5% normal donkey serum) for 1 hr. Primary antibodies in blocking solution were then added and incubated at 4°C for overnight, followed by washing and incubation with secondary antibodies. Cells were counterstained with DAPI before mounting.

The following antibodies were used: TUJ1 (Covance PRB-435P, rabbit or mouse, 1:20,000), MAP2 (Abcam ab5392, chicken, 1:5000), SOX2 (Santa Cruz sc-17320, goat, 1:1000), CTIP2 (Abcam, ab18465, rat, 1:300), SABT2 (Abcam ab92446, mouse, 1:300), TBR2 (Abcam ab31940, rabbit, 1:300); BRN2 (Santa Cruz sc-28594, rabbit, 1:200), TLX (Shi lab[20], rabbit, 1:1000), BrdU (Accurate Chemical & Scientific Corp OBT0030, Rat, 1:6000), Cleaved Caspase3 (Cell Signaling 9661, rabbit, 1:200).

Images were obtained with a Carl Zeiss LSM700 confocal microscope or Nikon Eclipse TE-2000-S microscope. Cortical layer thicknesses, brain organoid size and GFP fluorescence intensity of brain organoids were measured by ImageJ. For measuring the layer thickness, small rectangles were drawn to ensure two opposite sides to align with both layer marker dashed lines.

Calcium imaging

Brain organoids at day 45 of differentiation were partially dissociated and seeded on Matrigel-coated Ibidi μ-slide 8-well-chamber slides and allowed to grow for 3 days until calcium imaging was performed. Brain organoids were rinsed in artificial-cerebrospinal fluid (ACSF) (124 mM NaCl, 2.5 mM KCl, 26 mM $NaHCO_3$, 1 mM $MgCl_2$, 2 mM $CaCl_2$, 1.25 mM NaH_2PO4 and 10 mM D-glucose solution) at 37°C for 10 min and then incubated in fresh 95% O2 oxygenated ACSF containing 2 μM Fluo-4 AM for 20 min. Subsequently, brain organoids were visualized using a Zeiss Axio Observer Z1 microscope for serial time lapse imaging. Time lapse imaging was acquired at 10x magnification and at 16 frames per second speed for 5 min using a Hamamatsu EMCCD model C9100-13. Glutamate (3 μM) stimulation during imaging progress was performed 5 s after start. Ca^{2+} imaging videos were captured and processed using ZEN software and quantification was performed using Image-Pro Premier 9.1. Fluorescence intensity change over time is defined as: $\Delta F/F = (F - Fo)/Fo$, where F is the fluorescence intensity at any time point, and F_o is the baseline fluorescence intensity averaged across the whole movie for each cell. For calcium imaging of HCMV (TB40/E-Gluc without GFP)-infected brain organoids at

day 45 of differentiation, organoids were partially dissociated and allowed to attach overnight onto Matrigel-coated Ibidi μ-Slide 8 Well chamber slide. Brain organoids were either exposed to HCMV at 5 × 10^5 pfu/ml per brain organoid or to a mixture of HCMV and NAb 1B2. For NAb treatment, the same amount of virus was incubated with 4,000 ng/ml 1B2 antibody at 37°C for 1 hr before addition to the brain organoid culture. As a control, brain organoids were infected with 5 × 10^5 pfu/ml UV-inactivated HCMV. Calcium imaging was performed 3 days post infection. In order to identify cells that were infected by HCMV, calcium imaging data of each area were recorded with the associated coordinates using most bottom-right corner as a reference (0, 0). Right after calcium imaging, the brain organoids were immediately fixed with 4% PFA and immunostained for HCMV IE. For imaging, the fields were chosen based on the calcium imaging video coordinates and the positions were adjusted manually to match the original calcium imaging video pictures. By using this procedure, we were able to locate the HCMV positive cells by IE1 positive staining.

Microelectrode Arrays (MEA)

Brain organoids at day 45 of differentiation were partially dissociated and seeded onto 12-well transparent MEA plates at three brain organoids per well. Brain organoids were cultured in BrainPhys medium, including 1 × B27, 1 × N2, 20 ng/ml GDNF, 20 ng/ml BDNF, 500 μg/ml Dibutyryl-cAMP, 1 × Glutamax, and 1 × NEAA. MEA recordings were performed at 37°C in a Maestro MEA system with AxIS software using a bandwidth with a filter for 10Hz to 2.5 kHz cutoff frequencies. Spike detection was performed using an adaptive threshold set to 5.5 times of the standard deviation of the estimated noise on each electrode. For recordings, following a 5 min resting time in the Maestro instrument, each plate was recorded for 10 min to calculate the spike rate per well. When a recording of 5 spikes over the length of 1 min (5 spikes per min) was obtained, the electrode was considered active. Individual electrode bursts were identified using an adaptive Poisson surprise algorithm, while network bursts were identified for each well using a non-adaptive algorithm requiring a minimum of 10 spikes with a maximum inter-spike interval of 100 ms. Multielectrode data analysis was performed using the Axion Biosystems NeuralMetrics Tool. Synchrony index was calculated by NeuralMetric Tool with synchrony window set as 20 ms. For the pharmacological experiment, CNQX (10 μM) or bicuculline (10 μM) were applied to plate immediately before recording. For MEA recording, brain organoids treated with UV-irradiated TB40/E were included as the control organoids. Brain organoids were exposed to TB40/E or UV-irradiated TB40/E at 5 × 10^5 pfu/ml per brain organoid in the presence of 4000 ng/ml IgG. For NAb treatment, 5 × 10^5 pfu/ml of TB40/E was incubated with 4000 ng/ml NAb 1B2 for 1 hr and then added to brain organoids cultures. Wave forms of spike were generated from exported recording data on single electrode and the graph was created in Excel. The phase contrast and GFP fluorescent images of organoids seeded in the MEA plates were taken after MEA recording.

Generation of NPCs from human iPSCs

hiPSC-derived NPCs were generated according to previously described procedures[75]. Briefly, IMR90 hiPSCs were dissociated with Accutase into single cells and seeded onto a Matrigel-coated 6-well plate at 1 × 10^5 cells per well in E8 medium containing 1 μM Y-27632. On the next day, the E8 medium was substituted with NPC induction medium, including E6 medium, 100 nM ATRA, 10 μM SB431542, 250 nM LDN-193189, and the NPC induction medium was changed every day for 8 days. Cells were then transferred to a T25 or T75 flask and maintained in NPC maintenance medium containing 1 × B27, 1 × N2 supplement, 1 × NEAA, 1 × Glutamax, 100 nM ATRA, 3 μM CHIR99021, 2 μM SB431542, 10ng/ml EGF and 10ng/ml FGF, with daily medium change.

Neuronal differentiation from NPCs

Three million hiPSC-derived NPCs were dissociated with Accutase and seeded on Matrigel-coated 10 cm plates in neural induction medium containing DMEM/F12, 1 × N2, 1 × B27, 1 × NEAA, 1 × Glutamax. Cells were cultured in this medium for 3 weeks and then transferred to BrainPhys medium and maintained in the medium with medium change every 4-5 days.

RNA-sequencing

hiPSC-derived brain organoids at day 45 differentiation were infected with 5x10^5 pfu/ml TB40/E or UV-irradiated TB40/E as a control. Total RNA was isolated from TB40/E-infected organoids or control organoids 15 days post infection using Trizol. RNA quality control was performed by the Integrative Genomics Core at City of Hope. RNA-sequencing reads were aligned against the human genome (hg19) using TopHat2[76]. Read counts were quantified using htseq-count with UCSC known gene annotations. Aligned reads were counted using GenomicRanges. Genes were filtered to only include transcripts with RPKM values greater than 0.1 (after a rounded log2-transformation) in at least 50% samples. Genes smaller than 150 bp were removed prior to differential expression analysis. Log_2 (RPKM + 0.1) expression values were used for visualization and fold-change calculations. Normalization of heatmap values was performed as the following: RNA seq of control sample was converted by Log_{10} of the RPKM value, the HCMV infected sample value was determined by $sum = log10(RPKM\ value) + DEseq2 - log2(fold\ change\ value)$. Heatmap was generated by ClustVis and image was prepared using Photoshop.

RT-qPCR analysis

For siRNA knockdown of receptors in iPSC-derived NPCs, total RNA was extracted using Trizol Reagent. cDNAs were reverse transcribed using Tetro cDNA synthesis kit. RT-qPCR was performed using DyNAmo Flash SYBR Green qPCR mix on a StepOnePlus system and normalized to β-actin. Primers used in the qPCR are listed in Key Resources Table.

RNA interference

Oligonucleotides for siRNA-mediated RNA interference were synthesized by Integrated Device Technology. Oligonucleotides used for RNA interference were listed in the Key Resources Table. For RNA interference in brain organoids, four brain organoids with similar size were seeded in ultralow 24-well plates (one organoid per well) and transfected with siRNA using siLentFect. After 96 hr incubation, each organoid was infected with TB40/E at 5×10^5 pfu/ml. Medium was changed the following day. Brain organoids were cultured for 20 days on shaker with continuous medium change every 3-4 days.

Western blot analysis

Cells were lysed in 0.1 M Na_2CO_3 containing 2 mM PMSF, and cell lysates were sonicated using Sonic Dismembrator to disintegrate genomic DNA. Protein concentrations were measured by Bradford Assay. Forty μg total proteins of NPCs and neurons were loaded for western blot analysis. EGFR and PDGFRα antibodies were used at 1:1000 dilution.

Plasmids

EGFR WT was a gift from Matthew Meyerson (Addgene plasmid # 11011; http://addgene.org/11011; RRID:Addgene_11011). The pHIV7-EGFR-T2A-DsRed vector was generated by cloning the human EGFR coding region via SwaI and NotI sites into the plasmid pHIV-T2A-DsRed (kindly provided by Dr. Xiuli Wang from City of Hope), resulting in the EGFR expression vector. pDONR223-PDGFRA was a gift from William Hahn & David Root (Addgene plasmid # 23892; http://addgene.org/23892; RRID:Addgene_23892). The pcDNA-PDGFRα vector was created by cloning the human PDGFRα cDNA into pcDNA3.1(+) neo vector using NheI and SmaI sites. The sequences of the cDNA were verified by Sanger sequencing.

hiPSC electroporation and HCMV infection

Control DsRed plasmid DNA or the mix of EGFR-T2A-DsRed and PDGFRα plasmid DNAs were introduced into hiPSCs by electroporation using 4D-Nucleofector following manufacturer's instructions. Briefly, 3 million hiPSCs in single cell suspension were mixed with P3 solution and subjected to electroporation using program CA137. The electroporated iPSCs were immediately transferred onto Matrigel-coated 24-well plate in E8 medium containing 1μM Y27632. The hiPSCs were subcultured twice in order to remove dead cells and subsequently allowed to attach on a Matrigel-coated 24-well plate at $2x10^4$ cells/well. hiPSCs were infected with TB40/E at a MOI of 5 and harvested at 2 days after infection. The percent of CMV IE1-positive and DsRed-positive (CMV+DsRed+) cells out of total DsRed+ cells was quantified and plotted.

QUANTIFICATION AND STATISTICAL ANALYSIS

Statistical analysis

All statistical analyses were performed using GraphPad Prism7.0. Statistical details of experiments can be found in the figure legends. All data are shown as mean ± SD or SE. The statistical significance of experiment outcome when comparing two or more groups was calculated using two-way ANOVA followed by Tukey's, Dunnet's or Sidak's multiple comparison tests or Student's t test. The difference between experimental groups was considered significant when $p < 0.05$.

DATA AND CODE AVAILABILITY

The RNA-seq dataset generated during this study is available at NCBI. The GEO Accession Super Series ID is GEO: GSE145415.

Cell Reports Medicine, Volume *1*

Supplemental Information

Modeling Human Cytomegalovirus-Induced Microcephaly in Human iPSC-Derived Brain Organoids

Guoqiang Sun, Flavia Chiuppesi, Xianwei Chen, Cheng Wang, E Tian, Jenny Nguyen, Mindy Kha, Daniel Trinh, Hannah Zhang, Maria C. Marchetto, Hongjun Song, Guo-Li Ming, Fred H. Gage, Don J. Diamond, Felix Wussow, and Yanhong Shi

Supplemental Figures and Legends

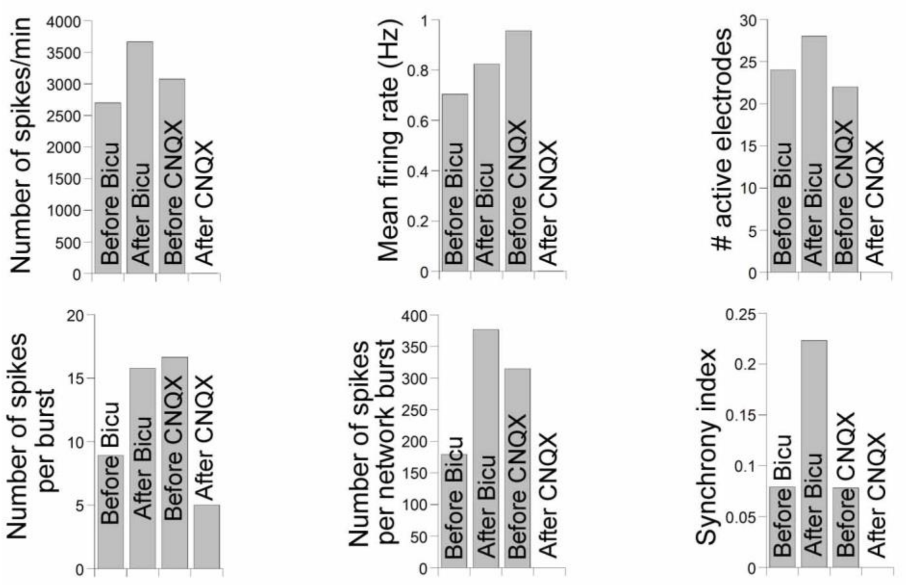

Figure S1. Functional neuronal networks shown by MEA recording. Related to Fig. 1. Quantification of MEA parameters in brain organoids in 10 min recording before or after treatment with GABAergic neuronal inhibitor bicuculine (Bicu), and before or after glutamatergic neuronal inhibitor CNQX treatments.

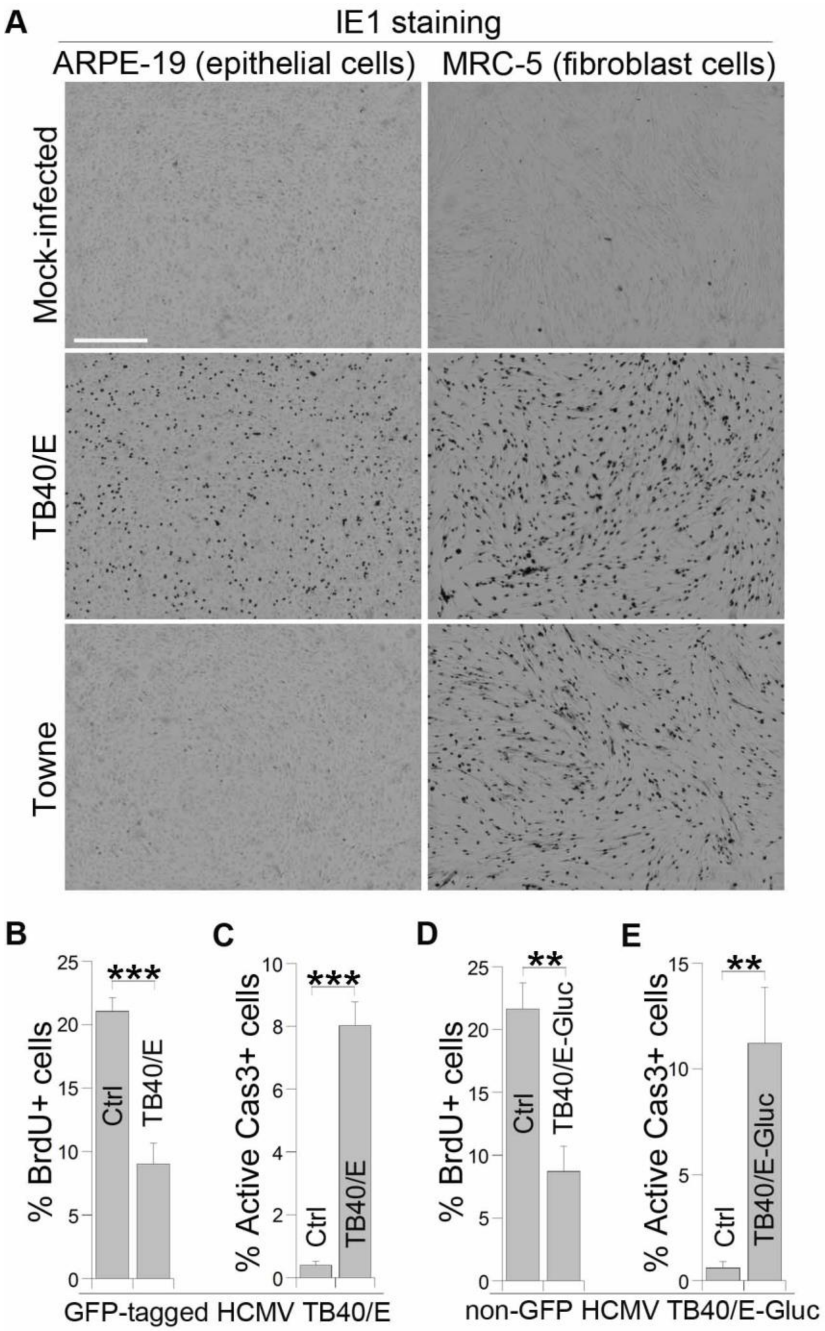

Figure S2. HCMV cell tropism and HCMV-induced abnormal cell proliferation and apoptosis in hiPSC-derived brain organoids. Related to Fig. 2. (**A**) ARPE-19 epithelial cells and MRC-5 fibroblasts were seeded in 96-well plates and either mock-infected or infected with TB40/E or Towne. HCMV-infected cells were immunostained for HCMV IE1 at 24 hours post infection. Shown are representative images of the IE1 immunostaining. Scale bar: 500 µm. (**B-E**) Quantification of the number of proliferating and apoptotic cells in brain organoids infected with the GFP-tagged HCMV TB40/E (B and C) or the non-GFP tagged HCMV TB40/E-Gluc (D and E) in comparison to mock-infected brain organoid controls. The number of proliferating cells was evaluated by the percent of BrdU+ cells out of total cells, while apoptosis was assessed by the percent of active caspase 3-positive (Active Cas3+) cells out of total cells. Bars represent mean ± SD. **p<0.01 and ***p<0.001 by Student's t-test . n=4 replicates.

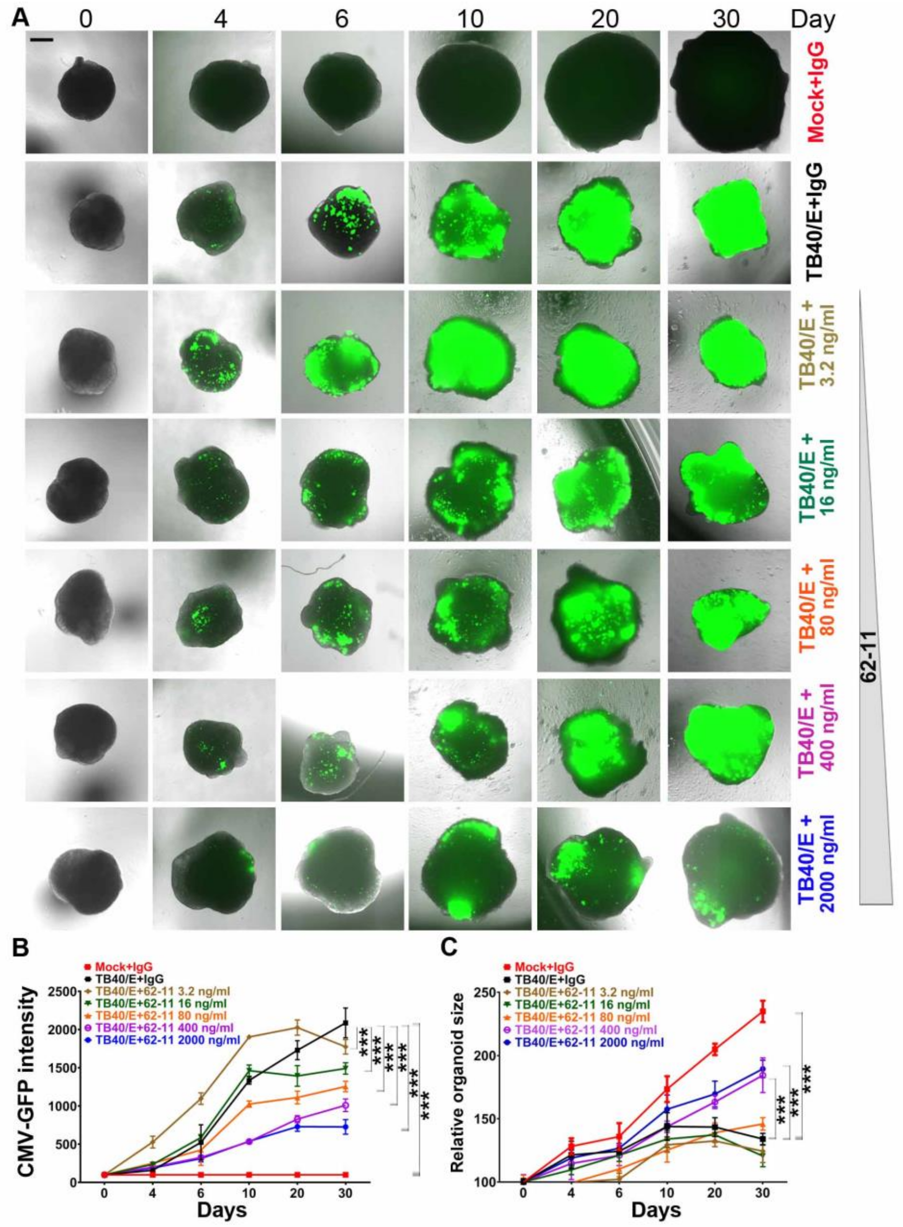

Figure S3. Prevention of HCMV TB40/E-induced abnormal brain organoid growth by Nab 62-11. Related to Fig. 3. hiPSC-derived brain organoids at day 45 of differentiation were infected with GFP-labeled TB40/E in the presence of different concentrations of NAb 62-11 that ranged from 3.2 ng/ml to 2000 ng/ml of antibody. Mock-infected brain organoids and TB40/E-infected organoids treated with IgG control (2000 ng/ml) were used as control organoids. **(A)** Representative images of control organoids and brain organoids infected with TB40/E in the presence of different concentrations of NAb 62-11. The images of the control organoids are the same as that in Fig. 3 because the experiments in Fig. 3 and Fig. S3 were performed in parallel. Scale bar, 200 µm. (**B, C)** Graphs illustrating the HCMV-GFP fluorescence intensity (B) and growth kinetics (C) of control organoids and TB40/E-infected brain organoids treated with NAb 62-11. Growth kinetics is measured using relative organoid size. The relative organoid size for each time point is given as the % of the organoid size (100%) at day 0 of infection. Values represent mean ± SD. *** p<0.001 by two way ANOVA followed by Tukey's multiple comparison test. n=4 organoids per group.

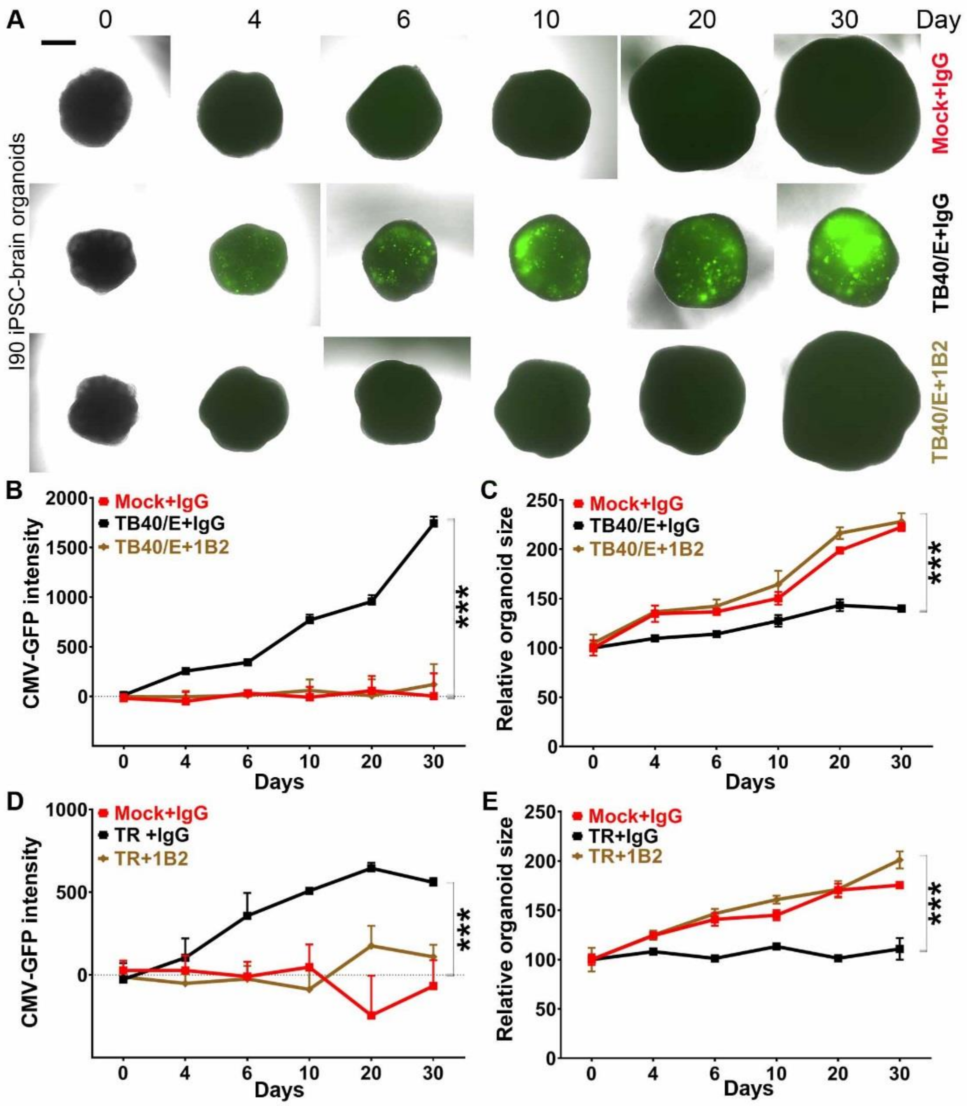

Figure S4. NAb prevents abnormal brain organoid growth induced by TB40/E and TR in hiPSC-derived organoids. Related to Fig. 3. Human IMR90 iPSC-derived brain organoids at day 45 of differentiation were infected with GFP-labeled TB40/E or TR in the absence or presence of NAb. Mock-infected brain organoids were used as a control. (**A**) Representative images of brain organoids that were mock-infected or infected with TB40/E in the presence of IgG (Mock + IgG, or TB40/E + IgG) or brain organoids infected with TB40/E in the presence of NAb 1B2 (TB40/E + 1B2) at the given time points during 30 days post infection. Scale bar, 200µm. (**B, C**) Graphs indicating the relative GFP fluorescence intensity (arbitrary units) (B) or relative organoid size (C) in mock-infected control brain organoids, or brain organoids infected with TB40/E in the absence or presence of NAb 1B2. (**D, E**) Graphs indicating the relative GFP fluorescence intensity (arbitrary units) (D) or relative organoid size (E) in mock-infected control brain organoids, or brain organoids infected with TR in the absence or presence of NAb 1B2. The relative organoid size for each time point is given as the % of the organoid size (100%) at day 0 of infection. Values represent mean ± SD. *** $p<0.001$ by two way ANOVA followed by Tukey's multiple comparison test. n=4 replicates.

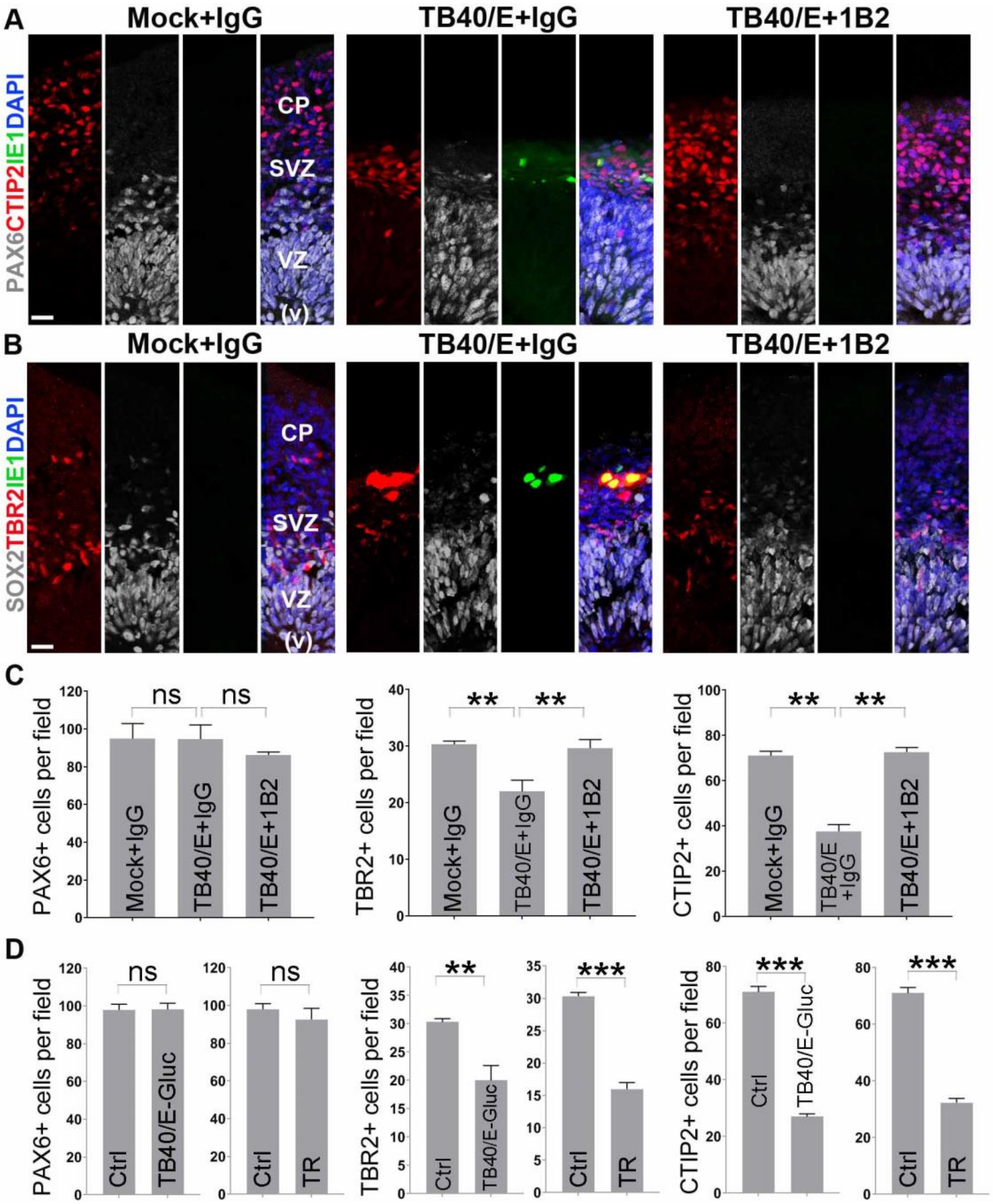

Figure S5. NAb prevents TB40/E-induced abnormal layer composition in brain organoids. Related to Fig. 4. (**A, B**) Representative images (cropped from images in Fig. 4C, D) were used for counting specific layer marker-positive cells. Scale bar, 20 µm. (**C**) Quantification of PAX6+, TBR2+, and CTIP2+ cells in brain organoids that were mock-infected in the presence of IgG control or infected with TB40/E in the presence of IgG control or NAb 1B2, in images shown in panels A and B. $p > 0.05$ (ns), $^{**}p < 0.01$ by one-way ANOVA followed by Tukey's multiple comparison test. n=4 replicates. (**D**) Quantification of PAX6+, TBR2+, and CTIP2+ cells in mock-infected control organoids and brain organoids infected with non-GFP-tagged TB40/E-Gluc or TR. $p > 0.05$ (ns), $^{**}p < 0.01$, $^{***}p < 0.001$ by Student's t-test. n=4 replicates. For panels C & D, values represent mean ± SD.

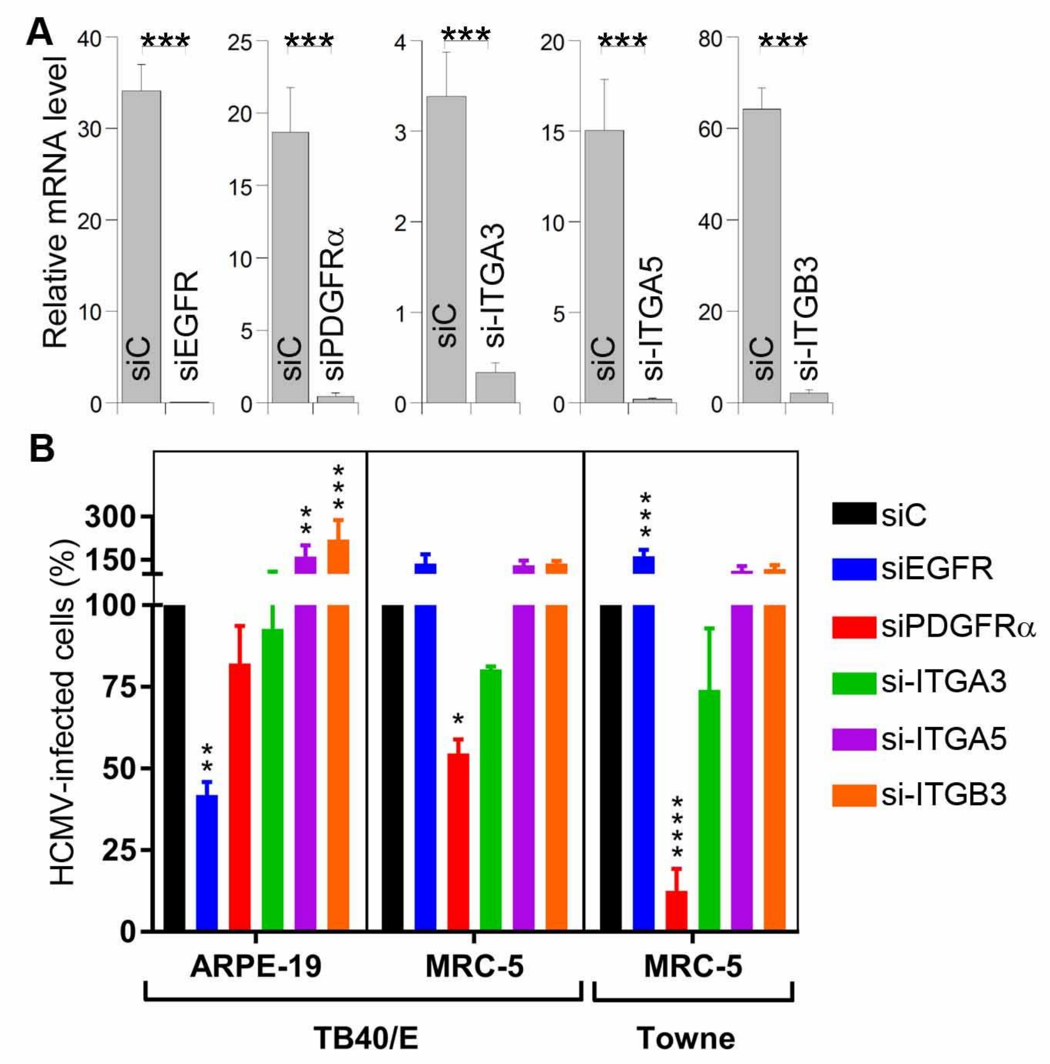

Figure S6. Knockdown of receptor gene expression by receptor-specific siRNAs. Related to Fig. 5. (A) Evaluation of siRNA efficacy. hiPSC-derived NPCs were transfected with siRNAs specific for PDGFRα, EGFR, ITGA5, ITGB3, ITGA3, or non-targeting control siRNA (siC). At four days post transfection, mRNA expression of specific genes was analyzed by RT-qPCR. Values represent mean ± SD. ***p < 0.001 by Student's t-test. n=4 replicates. (**B)** siRNA-mediated inhibition of HCMV infection of fibroblast and epithelial cells. ARPE-19 and MRC-5 cells were treated with the receptor-specific siRNAs or siC. Following 48-hour incubation, cells were infected with TB40/E or Towne and stained for HCMV IE1 at 24 hours post-infection. The percent of HCMV-infected cells was calculated relative to the number of IE1-positive cells in HCMV-infected cells treated with siC. Values represent mean ± SEM of three independent experiments performed in triplicate wells. Statistical significance was calculated to HCMV/siCTRL group using 2-way ANOVA followed by Dunnett's multiple comparisons test. *p<0.05, **p<0.01, ***p<0.001, and ****p<0.0001. n=3 replicates.

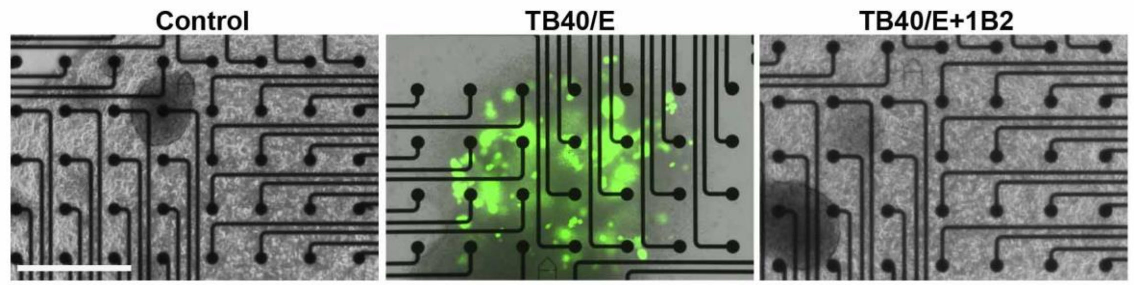

Figure S7. **Representative images of brain organoids seeded on MEA electrodes. Related to Fig. 7.** Sample images of mock-infected brain organoids and brain organoids infected with GFP-tagged TB40/E in the absence or presence of 1B2 NAb are shown. Scale: 500 µm.
